# Therapeutic hepatitis B vaccine employing DNA prime – MVA boost scheme requires additional priming with recombinant HBsAg to elicit an adequate antibody response

**DOI:** 10.3389/fimmu.2026.1771887

**Published:** 2026-03-12

**Authors:** Hélène A. Kerth, Martin Kächele, Nikolas Müller, Swati Singh, Margaret Tulessin, Julia Sacherl, Edanur Ates Öz, Jinpeng Su, Romina Bester, Susanne Miko, Katja Steiger, Carolin Mogler, Dirk H. Busch, Jochen M. Wettengel, Ulrike Protzer, Anna D. Kosinska

**Affiliations:** 1 Institute of Virology, School of Medicine and Health, Technical University of Munich/Helmholtz Munich, Munich, Germany; 2 German Center for Infection Research (DZIF), Munich Partner Site, Munich, Germany; 3 Institute of Pathology, School of Medicine and Health, Technical University of Munich, Munich, Germany; 4 Institute for Medical Microbiology, Immunology and Hygiene, School of Medicine and Health, Technical University of Munich, Munich, Germany

**Keywords:** DNA vaccine, functional cure, HBsAg seroconversion, HBV, hepatitis B, therapeutic vaccine, *TherVacB*

## Abstract

Therapeutic vaccines offer hope for a curative treatment of chronic hepatitis B virus (HBV) infection. We developed a therapeutic hepatitis B vaccine, *TherVacB*, which employs priming with adjuvanted HBV S (HBsAg) and Core antigens of a single genotype, and a boost with a pan-genotypic Modified Vaccinia Ankara vector (MVA-HBVac) expressing several viral proteins that cover more than 95% of HBV circulating strains. Priming immunization with antigens exactly matching those expressed by the MVA may improve the efficacy of *TherVacB*. However, recombinant protein antigens need to be produced separately and require complex and costly purification, as well as a Th1-inducing adjuvant, to elicit CD8^+^ T-cell responses. These limitations may be overcome by DNA vaccines, which enable the direct expression of several antigens *in vivo*. Here, we investigated the potential of DNA vaccination as an alternative to protein priming to broaden and enhance HBV-specific immunity. Immunization of HBV-carrier mice with a DNA prime-MVA boost regimen elicited robust HBV-specific CD8^+^ T-cell responses, capable of reducing serum HBeAg levels and eliminating HBV-infected hepatocytes. However, it yielded poor neutralizing anti-HBs titers, resulting in an inefficient reduction of circulating HBsAg. Including adjuvanted HBsAg into DNA priming, either simultaneously or sequentially, significantly enhanced HBsAg-to-anti-HBs seroconversion rates. Still, only the sequential application supported vigorous plasmid-mediated CD8^+^ T-cell immunity. Interestingly, sequential immunization with DNA, followed by HBsAg and MVA, elicited neutralizing anti-HBs antibodies and enhanced HBV-specific CD8^+^ T-cell responses, resulting in a more potent antiviral effect compared to the reverse priming regimen. Our study demonstrates that although DNA can successfully prime an effective antiviral CD8^+^ T-cell response, it requires additional immunization with adjuvanted HBsAg to elicit satisfactory neutralizing antibody titers. In addition, it demonstrates that the sequence of immunizations in multicomponent heterologous-prime boost regimens is crucial and can significantly affect the quantity and quality of induced immune responses.

## 1 Introduction

Despite access to safe and effective prophylactic vaccines for over 40 years, hepatitis B virus (HBV) infection represents a significant global health burden, with an estimated 250 million people worldwide suffering from chronic hepatitis B (CHB) (1). Treatment of CHB with currently available antivirals is rarely curative (2), thus leaving these individuals at high risk of developing life-threatening liver cirrhosis and hepatocellular carcinoma. Since an estimated 1.1 million people die every year due to HBV-related complications (1), development of a broadly applicable, finite therapy of CHB represents an urgent medical need. It constitutes one of the World Health Organization’s primary goals in eliminating viral hepatitis by 2030 (3).

It is thoroughly documented that progression toward persistent HBV infection is associated with impaired innate and adaptive HBV-specific immunity (4, 5). Chronic HBV carriers develop HBV-specific immune tolerance, characterized by the absence of anti-HBs antibodies and weak or absent virus-specific CD4^+^ and CD8^+^ T-cell responses (6). Thus, therapeutic vaccines that reactivate HBV-specific immunity in chronically infected patients, thereby eliminating HBV-infected hepatocytes, represent a promising approach to cure CHB. To this end, we developed *TherVacB*, a clinical candidate therapeutic hepatitis B vaccine that follows a heterologous prime-boost scheme. Adjuvanted recombinant surface and core antigens (HBsAg and HBcoreAg), which form virus-like particles, are used for priming, whereas the modified vaccinia virus Ankara (MVA) vector expressing HBV antigens is used as a boost (7). In preclinical mouse models, we demonstrated that *TherVacB* simultaneously elicited strong anti-HBs, CD4^+^, and CD8^+^ T-cell responses against HBV, resulting in efficient neutralization of HBV in the blood, elimination of infected hepatocytes, and immune control of persistent replication (8, 9). To facilitate clinical translation and broaden *TherVacB*-induced HBV-specific immune responses to the most prevalent HBV genotypes (gt) and serotypes, we generated a novel MVA vector, MVA-HBVac, which expresses optimized sequences of HBV Core, small and large envelope proteins (S and L), and the reverse transcriptase domain of polymerase [RT(pol)] covering >95% of HBV strains circulating worldwide (10). When administered as a booster vector in an established *TherVacB* vaccination regimen, following two primes with adjuvanted HBsAg of gtA and HBcoreAg of gtD, MVA-HBVac elicited strong HBV-specific immunity and reduced the viral parameters in mice replicating HBV of genotypes A, B, and D (11). Since successful *TherVacB* vaccination largely depends on optimal priming (12), we reasoned that a more complementary prime immunization, encompassing multiple HBV antigens of the most commonly circulating strains, combined with the MVA-HBVac boost, may further enhance the vaccine’s immunogenicity. In addition, it would allow priming of immune responses against the HBV polymerase and the pre-S domain of the large (L) envelope protein, which are included in the MVA-HBVac boost but not covered by the priming protein antigens.

Protein vaccines require an optimized production protocol for each antigen, which includes complex and challenging purification steps. This is time-consuming and becomes costly when multiple antigens of interest are needed. In addition, to be used in therapeutic settings, they require particular adjuvants to elicit potent effector CD8^+^ T cell responses (12). As an alternative, DNA vaccines induce endogenous production of the antigens of interest *in vivo*, representing a cost-effective, easy-to-upscale vaccine platform that might be especially useful for challenging-to-produce antigens, such as RT(pol) (13). Moreover, DNA vaccines feature intrinsic adjuvanticity through unmethylated CpG motifs, triggering innate immune system activation through TLR-9 signaling (14). Despite limited immunogenicity in clinical trials, DNA-based vaccines have still shown improved efficacy when employed for priming, in a heterologous prime-boost regimen, followed by a boost immunization with a viral vector encoding the same antigens (15–17).

In this study, we aimed to investigate whether a pan-genotypic DNA vaccine could successfully substitute adjuvanted recombinant proteins of a single genotype in *TherVacB* priming and improve vaccine efficacy when combined with the new MVA-HBVac vector for boosting. Thus, we generated DNA-HBVac, a plasmid encoding the optimized sequences of HBV proteins [Core, S, L, and RT(pol)], that exactly match the expression cassette of MVA-HBVac. The immunogenicity of the DNA prime – MVA boost *TherVacB* regimen was assessed in HBV-naïve and HBV-carrier mice transduced with AAV-HBV and compared with the classical *TherVacB* regimen.

## 2 Materials and methods

### 2.1 Generation of DNA-HBVac

DNA-HBVac was generated based on the pVAX plasmid (Invitrogen, Karlsruhe, Germany). It contains cytomegalovirus (CMV) immediate early (IE) promoter (CMV-IE), followed by the R region from human T cell leukemia virus type 1 (HTLV-1) (with a splice donor) and the CMV immediate-early 3’ intron (with a splice acceptor) (18), the inserted HBVac cassette, and the BGH polyadenylation signal (polyA). The HBVac insert encodes for five HBV proteins: the small envelope protein S (subtype adw, gtA), the large envelope protein L (ayw, gtC), the full-length core protein forming the HBV capsid Core_1-183_ (gtC), a C-terminally truncated version of the Core protein Core_1-149_ (gtD), and a consensus sequence of the reverse transcriptase (RT) domain of the viral polymerase (pol), without eliminating its enzymatic activity. The sequences were optimized to cover the known HBV epitopes while remaining identical to naturally occurring viral sequences to ensure correct folding and processing (11). For efficient protein synthesis, the Kozak sequence (GCC ACC) was inserted before the ATG codon of the first HBVac protein (S). Codon-optimized porcine teschovirus-1 2A (P2A) and thosea asigna virus 2A (T2A) (19, 20) were inserted between the sequences of each HBV protein. Thus, the natural stop codons between the individual HBV proteins were removed.

The HBVac insert was chemically synthesized (Invitrogen, Karlsruhe, Germany) and delivered in a transfer plasmid. The HBVac insert was amplified via PCR using the following primers (Fw: 5’-CGGTACCGTCGACACGTGGGCGCGCCAGATCTGAGC-3’; Rev: 5’- TCTAGATGATCACACGTGTTAATTAAAGATCTAAGCTTACGCG-3’), purified by gel electrophoresis, and then integrated into the multiple cloning site of the plasmid. The correct generation of DNA-HBVac was verified by restriction digestion and Sanger sequencing. DNA-HBVac was amplified in chemically competent *E. coli* (stlb3, Invitrogen, Karlsruhe, Germany) and purified using lipopolysaccharide (LPS)-free NucleoBond Xtra Midi Kit (Machery-Nagel, Düren, Germany).

### 2.2 Cell culture and DNA transfection

HepG2 (ATCC^®^ HB-8065) and HepG2-NTCP cells (21) were cultured in DMEM medium (GIBCO™, Karlsruhe, Germany) enriched with 10% FCS (Thermo Fisher Scientific Inc., Waltham, USA), 1% penicillin/streptomycin (10.000 U/ml), 1% non-essential amino acids (100x), 200 mmol/L L-glutamine, 0,2% HEPES, and 1% sodium pyruvate (all from GIBCO™). One day prior to the transfection experiments, HepG2 cells were seeded at 1.5 × 10^5^ per well in 12-well plates. DNA transfection was performed according to the manufacturer’s protocol using Lipofectamine 2000 (Invitrogen, Carlsbad, CA, USA) and 1µg DNA-HBVac plasmid. Mock-transfected cells served as controls.

### 2.3 Western blotting and HBV antigen detection

HepG2 cell lysates were obtained 24 h post-transfection using Pierce RIPA buffer (Thermo Scientific Fisher) supplemented with a cOmplete protease inhibitor (Roche, Mannheim, Germany). Proteins were separated by SDS-PAGE and transferred to Polyvinylidene difluoride (PVDF) membranes (Amersham, Slough, United Kingdom). Unspecific antibody binding was blocked in 5% milk (Sigma-Aldrich, Taufkirchen, Germany). Expression of HBV proteins was analyzed using the following primary antibodies: mouse anti-Core (8C9, in-house generated), mouse anti-S (HB1, kindly provided by D. Glebe, Justus Liebig University Giessen, Germany), rabbit anti-L (H863, kindly provided by S. Urban, University Hospital Heidelberg, Germany), and rat anti-RT(pol) (clone 9H2, kindly generated by Helmholtz Monoclonal Antibodies Core Facility, Munich). Proteins were detected using a horse radish peroxidase (HRP) coupled secondary antibody and ECL Prime Western Blotting Detection Reagent (GE Healthcare, Freiburg, Germany). HBsAg was detected in the HepG2 supernatant 24 h and 48 h post DNA-HBVac transfection on Cobas^®^ e411 analyzer (Roche Diagnostics, Basel, Switzerland), while HBcoreAg was quantified by in-house sandwich ELISA as previously described (22). Cell viability was assessed using the CellTiter-Blue^®^ assay (Promega, Madison, WI, USA) according to the manufacturer’s instructions.

### 2.4 Ethical statement

Animal experiments were rigorously performed according to the European Health Law of the Federation of Laboratory Animal Science Associations (FELASA), the German Society for Laboratory Animal Science GV-SOLAS, and the principles of the 3Rs (Replacement, Reduction, and Refinement). The experiments were approved by the Committee of Upper Bavaria (permission number: ROB-55.2-2532.Vet_02-18-24). Animals received care according to the criteria listed in the “Guide for the Care and Use of Laboratory Animals” issued by the National Academy of Sciences (NIH publication 86–23 revised 1985). Mice were kept in a biosafety level 2 pathogen-free animal facility at the Technical University of Munich. All experiments were completed during daylight.

### 2.5 Animals and AAV-HBV infection

Nine-week-old wildtype C57BL/6JRj male mice (haplotype H-2^b/b^) were acquired from Janvier Laboratories (Le Genest-Saint-Isle, France). To establish persistent HBV replication in wildtype C57BL/6JRj male mice, animals were intravenously (i.v.) transduced with 4-6 × 10^9^ genome equivalents of the adeno-associated virus (AAV)-HBV1.2 vector encoding for 1.2-overlength HBV genome of genotype D, serotype ayw (23). Before vaccination, mice were assigned to groups with comparable serological levels of HBsAg and HBeAg.

### 2.6 Heterologous prime-boost immunization

Animals were vaccinated in 2-week or 4-week intervals according to the previously reported *TherVacB* immunization protocol (11). For protein priming, mice received an intramuscular (i.m.) injection consisting of 10μg each of HBsAg (gtA, adw; Biovac, South Africa) and HBcoreAg (gtD, ayw; kindly provided by APP Latvijas Biomedicinas, Riga, Latvia), adjuvanted with 15µg of CpG-1018 (InvivoGen, San Diego, CA). For DNA priming, mice received i.m. injection with 50 µg or 100 µg of DNA-HBVac. Boosting immunizations were performed with 3 × 10^7^ infectious units (IFUs) of recombinant MVA-HBVac vector. The vector was amplified and purified as described previously (11).

### 2.7 Serological analyses

Serum levels of HBsAg, HBeAg, and anti-HBs antibodies were quantified using the Architect™ platform (Abbott Laboratories, Wiesbaden, Germany). Anti-HBc antibodies were detected using Liaison^®^XL Murex anti-HBc assay (DiaSorin, Saluggia, Italy) after diluting the mouse sera 1:10 in PBS. Enzymatic serum activity of alanine aminotransferase (ALT) was assessed in freshly prepared mouse serum (1:4 dilution in PBS) using Reflotron^®^ GPT/ALT stripe tests (Roche Diagnostics, Basel, Switzerland).

### 2.8 Histology and immunohistochemistry

Liver tissue samples were fixed in 4% buffered formalin for 48 h and embedded in paraffin. H&E staining was performed on deparaffinized sections with eosin and Mayer’s hemalum according to standardized protocols. Histopathologic evaluation was performed by an experienced liver pathologist. A general description of histopathologic findings, as well as scoring of inflammation based on Desmet grading of hepatitis (24) adapted to mice, which do not have predominant portal tract inflammation, were provided. Immunohistochemistry was performed using a rabbit anti-HBV core (#RP 017; 1:50 dilution; retrieval at 100 °C for 30 minutes with EDTA; Diagnostic Biosystems, Pleasanton, CA) on a Leica Bond MAX system (Leica Biosystems, Nussloch, Germany). HBcore-positive hepatocytes were identified in 10 random view fields (at 20x and 40x magnification) and quantified per square millimeter (mm^2^).

### 2.9 HBV neutralization assay

HepG2-NTCP cells were seeded at 70-80% confluency in cell culture medium supplemented with 2.5% DMSO (Merck, Darmstadt, Germany) 3 days before infection with purified reporter HBV expressing NanoLuc (rHBV) as previously described (25, 26). Mouse sera were pooled for each vaccination group, serially diluted in cell culture medium supplemented with 2,5% DMSO and 4% PEG-6000 (Merck, Darmstadt, Germany) containing rHBV reporter virus and incubated for 30 minutes at room temperature. Cells were inoculated overnight, then washed once with PBS and further cultured in a fresh cell culture medium supplemented with 2.5% DMSO. Cell culture media was exchanged on day 4 post-infection and supernatants were finally collected on day 7 post-infection. The luminescence measurement was performed using an Infinite F200 Tecan plate reader with 10 μl of supernatant and 100 μl of substrate solution (1 μM coelenterazine dissolved in acidified methanol diluted 1:1000 in PBS supplemented with 0.1% Tween-20; PJK, Kleinblittersdorf, Germany). Inhibition of infection was normalized to the linear range of the signal detected in naïve mouse sera. Normalized values were fit with a four-parameter nonlinear regression inhibitor curve in GraphPad Prism version 10.5.10 to obtain the half-maximal inhibitory concentration of infectivity (IC50).

### 2.10 Detection of HBV-specific T cells by multimer analysis and intracellular cytokine staining

Murine splenocytes and liver-associated lymphocytes (LALs) were isolated using a previously described protocol (27). Cell suspensions were stained with MHC class I multimers labeled with fluorescent Streptactin (APC or PE; IBA Lifesciences, Göttingen, Germany) as previously reported (28). To detect HBV-specific CD8 T cells, HBV S- [S_190_ (VWLSAIVM)] or core-specific [C_93_ (MGLKFRQL)] multimers were used, whereas control staining was performed with ovalbumin-specific [OVA_S8L_ (SIINFEKL)] multimers. For ICS, splenocytes and LALs were stimulated overnight at 37 °C with 1µg/ml of synthetic peptides in the presence of 1µg/ml of brefeldin A (Sigma-Aldrich, Taufkirchen, Germany). For HBV-specific stimulation, single S- and Core-derived CD8 T-cell epitopes: S_190_, S_208_ (IVSPFIPL), and C_93_, as well as optimized S-, Core-, and RT- derived overlapping peptide pools were used (11). Cells stimulated with MVA_B8R_ and OVA_S8L_ peptides served as positive and negative controls, respectively. Dead cells were excluded using fixable viability dye (eFluor780, eBioscience, Frankfurt, Germany), and extracellular staining of the cells was performed using anti-CD4-APC (clone GK1.5, eBiosciences) and anti-CD8a-PacificBlue (clone 53-6.7, BD Pharmingen, Allschwil, Switzerland) antibodies. Following fixation and permeabilization of the cells using Cytofix/Cytoperm Kit (BD Biosciences, Heidelberg, Germany), ICS was performed with anti-IFNγ antibody (clone XMG1.2, BD Biosciences). Absolute cell numbers were determined using CountBright™ counting beads (Thermo Fisher Scientific, USA) according to the manufacturer’s instructions. Samples were acquired on a CytoFlexS flow cytometer (Beckman Coulter, Brea, CA, USA), and analysis was performed using FlowJo version 10 software (Tree Star, Ashland, OR, USA).

### 2.11 Statistical analysis

Analysis was performed using GraphPad Prism software, versions 9.0.2 or 10.5.0 (GraphPad Software, Inc., San Diego, CA, USA). All graphs indicate mean ± SEM. Statistical analysis was conducted using nonparametric One-Way ANOVA with Kruskal-Wallis post-test, Mann-Whitney, or unpaired *t*-test. *P*-values < 0.05 were considered statistically significant and marked with asterisks.

## 3 Results

### 3.1 Generation of a DNA vaccine for TherVacB priming

**Figure 1:**
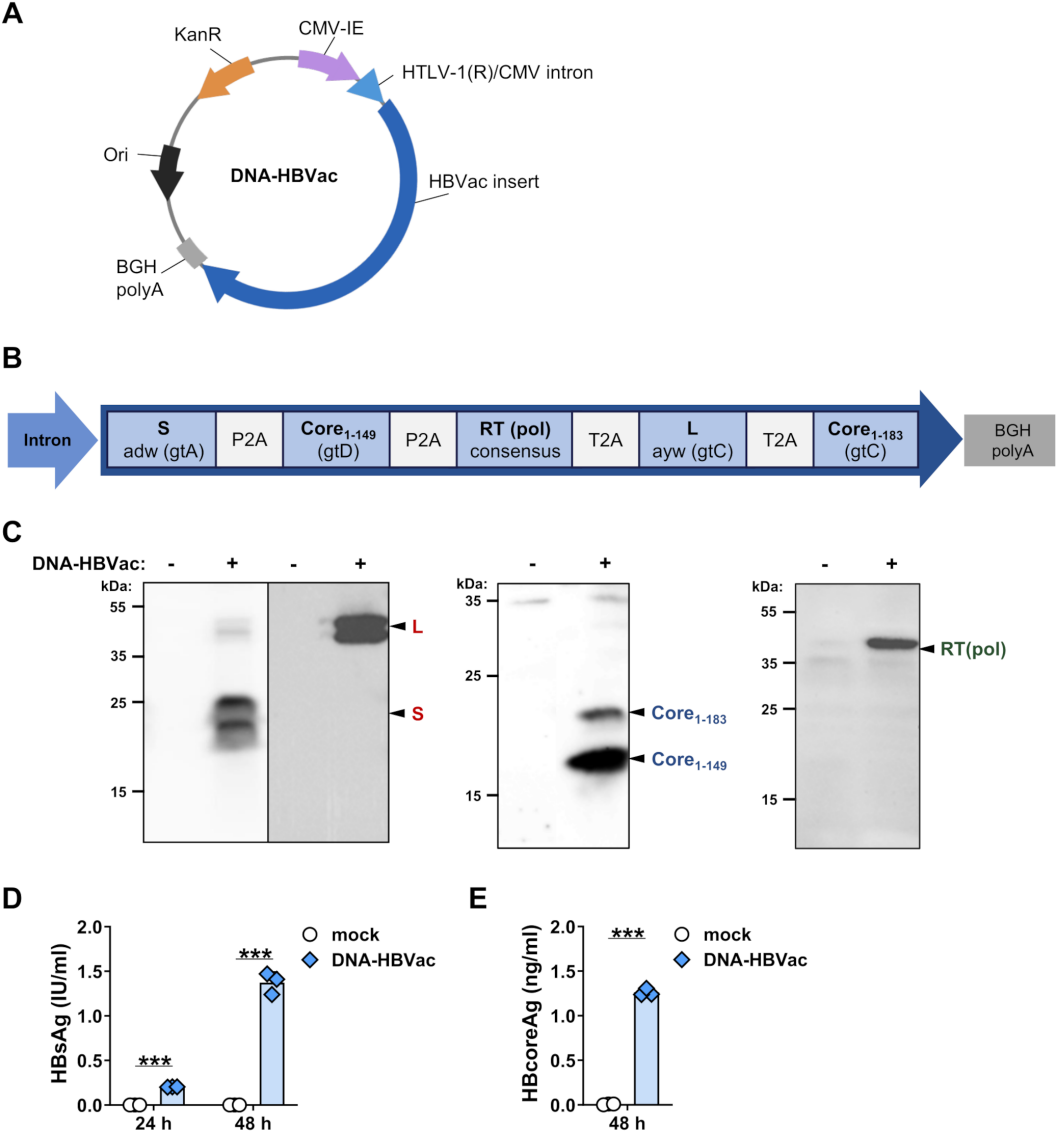
Generation of plasmid DNA vaccine: DNA-HBVac. **(A)** Schematic representation of DNA-HBVac, which drives the expression of HBVac cassette under the control of cytomegalovirus immediate early promoter (CMV-IE). It contains the regulatory (R) region from human T cell leukemia virus type 1 (HTLV-1) (with a splice donor), and the CMV immediate-early 3’ intron (with a splice acceptor) to improve antigen expression. **(B)** HBVac insert encodes for five HBV proteins: S, subtype adw, gtA; truncated Core_1-149_, gtD; RT domain of the viral polymerase [RT(pol)]; consensus sequence of L, subtype ayw, gtC; full-length Core_1-183_, gtC, linked by P2A and T2A sites. **(C)** Expression of all proteins was confirmed by Western blotting of HepG2 cell lysates 24 h post-transfection with 1 µg of DNA-HBVac. S and L proteins were detected at 24 kDa and at 42 kDa, respectively, in native and glycosylated forms. Truncated and full-length Core proteins were detected at 17 kDa and 21 kDa, respectively; RT(pol) was detected at 37 kDa. **(D)** HBsAg was detected in the supernatant of HepG2 cells at 24 and 48 h **(D, E)** Mean ± SD is indicated. Statistical analysis was conducted using unpaired t-test. *p*-values < 0.05 were considered statistically significant and marked with asterisks (***p < 0.001). KanR – kanamycin resistance gene, Ori-origin of replication, gt-genotype.

To investigate whether a DNA vaccine could be a suitable alternative to adjuvanted particulate proteins for *TherVacB* priming, we generated DNA-HBVac, which expresses the HBVac cassette under the control of the CMV-IE promoter (**Figure 1A**). To enhance the antigen expression and immunogenicity of DNA-HBVac, the sequences of the R region of the 5’ long terminal repeat of HTLV-1 and the CMV-IE 3’ intron, which serve as a pair of splicing signals (18), were inserted between the CMV-IE and the HBVac cassette. The HBVac insert encodes for five HBV proteins: the small envelope protein S (subtype adw, gtA), the large envelope protein L (ayw, gtC), the full-length core protein forming the HBV capsid Core_1-183_ (gtC), a C-terminally truncated version of the Core protein Core_1-149_ (gtD), and a consensus sequence of the reverse transcriptase (RT) domain of the viral polymerase (pol), which was designed based on consensus sequences of various genotypes (**Figure 1B**). The sequence of the HBVac insert was optimized not only to encompass T-cell epitopes of the most prevalent HBV genotypes A-E, which represent 95% of circulating isolates worldwide (10), but also to include serotypes adw and ayw (29). Codon-optimized P2A and T2A sites (19, 20) were inserted between the sequences of each HBV protein into the HBVac cassette. This allows equimolar translation of the individual proteins from a single mRNA via ribosomal skipping. When a ribosome encounters the specific motif within the P2A/T2A sequence, it releases the upstream protein and ‘skips’ to the next codon without the formation of a peptide bond to allow the synthesis of the downstream protein within the same translational complex (30).

To verify whether all encoded HBV antigens are correctly expressed, we transfected HepG2 cells with DNA-HBVac. We performed Western blot analysis 24 hours later using antibodies specific to S, L, Core, and RT(pol). We detected specific bands corresponding to HBV S and L proteins, in both glycosylated and non-glycosylated forms, as well as to the truncated and full-length core proteins, and to the RT domain of the viral polymerase (**Figure 1C**), confirming the successful generation of DNA-HBVac. Transfection of HepG2 cells with DNA-HBVac also resulted in the secretion of expressed antigens into the supernatant. The amount of secreted HBsAg increased at 48 hours post-transfection compared with the 24-hour time point, but remained at relatively low levels (**Figure 1D**). Also, HBcoreAg could be detected in the supernatant of transfected cells by ELISA (**Figure 1E**). No toxic effects of the transfection were observed (*data not shown*).

### 3.2 Immunogenicity of priming immunization with DNA-HBVac in HBV-naïve mice

**Figure 2:**
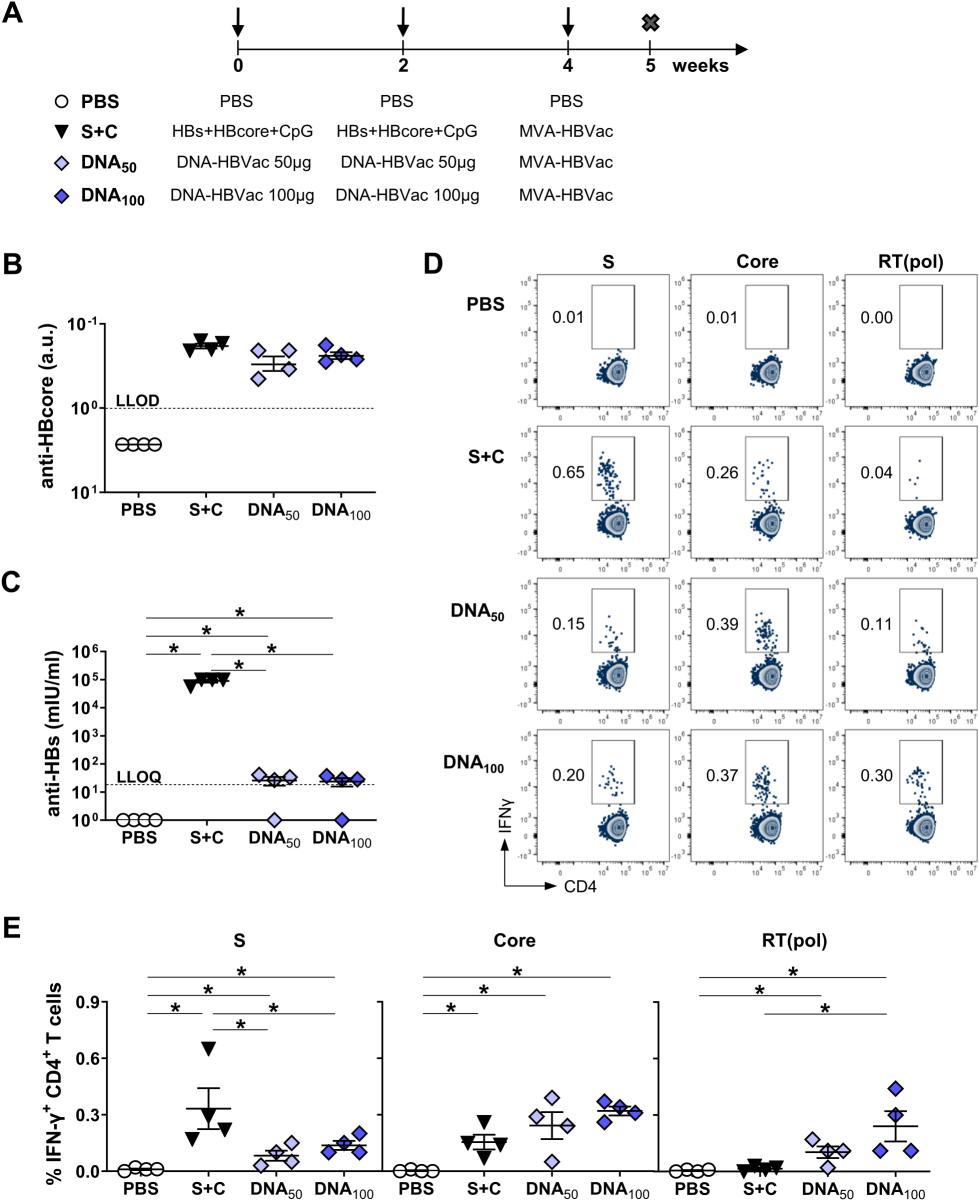
HBV-specific humoral and S-specific CD4^+^ T-cell responses induced by DNA prime – MVA boost immunization in HBV-naïve mice. **(A)** C57BL/6J mice (groups *n* = 4) were immunized twice in a two-week interval with 50 µg of DNA-HBVac (DNA_50_), 100 µg of DNA-HBVac (DNA_100_), or a mixture of 10 µg each of recombinant HBsAg and HBcoreAg adjuvanted with 15 µg of CpG (S+C), followed by a booster immunization with recombinant MVA-HBVac (3 × 10^7^ IFU/mouse) at week 4. Mice receiving PBS injections served as the control group (PBS). Mice were analyzed one week after boost immunization (week 5), at the peak of effector immune response. Serum levels of **(B)** anti-HBcore and **(C)** anti-HBs. **(D)** Representative flow cytometry plots of HBV-specific IFN-γ^+^ CD4^+^ T cells detected by intracellular cytokine staining (ICS) of splenocytes stimulated with HBV S-, Core-, and RT(pol)-specific overlapping peptide pools. **(E)** Frequencies of HBV S-, Core-, and RT(pol)-specific IFN-γ^+^ CD4^+^ T cells detected in splenocytes by ICS after *ex vivo* stimulation with corresponding peptide pools. **(B, C, E)** Mean ± SEM is indicated. Statistical analysis was conducted using nonparametric One-Way ANOVA. *p*-values < 0.05 were considered statistically significant and marked with asterisks (*p < 0.05). a.u.- arbitrary units; LLOD– lower limit of detection; LLOQ– lower limit of quantification.

To determine whether DNA-HBVac is immunogenic *in vivo*, we immunized HBV-naïve mice using the modified DNA prime–MVA boost *TherVacB* protocol and compared the induced immune response with that elicited by the ‘classical’ priming regimen, which uses recombinant HBV antigens. Three groups of C57BL/6 mice received two intramuscular immunizations with 50 μg or 100 μg DNA-HBVac (DNA_50_; DNA_100_), respectively, or with a mixture of adjuvanted particulate HBs and HBcore antigens (S+C) at weeks 0 and 2. Recombinant MVA-HBVac, encoding the same HBVac insert as DNA-HBVac and expressing the same set of proteins, served as a heterologous booster vaccine for all groups at week 4 (**Figure 2A**). Mice receiving intramuscular PBS injections (PBS) served as controls. Endpoint analyses were performed one week after the boost.

We first compared *TherVacB*-induced antibody response in mice receiving either DNA or recombinant proteins for priming. All mice developed significant anti-HBcore responses irrespective of the *TherVacB* regimen (**Figure 2B**). In contrast to DNA-HBVac priming, priming with adjuvanted antigens also induced high anti-HBs antibody titers reaching up to 10^5^ mIU/ml (**Figure 2C**). Levels of anti-HBs detected in mice primed with either 50 or 100 µg of DNA were, on average, 3 log_10_ lower than those detected after immunization with recombinant proteins.

Since efficient activation of CD4^+^ T cells during the priming phase of *TherVacB* strongly influences the vaccine-induced antibody and effector CD8^+^ T-cell responses, we investigated whether DNA priming elicits HBV-specific CD4^+^ T-cell responses using splenocytes. We found that both DNA-HBVac priming regimens, DNA_50_ and DNA_100,_ induced S-, core- and RT(pol)-specific IFN-γ CD4^+^ T-cell responses after *TherVacB* (**Figures 2D,E**). Priming with DNA-HBVac resulted in a significantly weaker S-specific IFN-γ CD4^+^ T-cell response compared to the adjuvanted protein prime, but especially at a 100µg dose, strong RT(pol)-specific IFN-γ CD4^+^ T-cell response.

We then examined whether priming with DNA-HBVac could trigger a strong HBV-specific CD8^+^ T-cell response, a key prerequisite for successful therapeutic vaccination. First, we determined the frequencies of S-specific IFN-γ^+^ CD8^+^ T cells in the spleen of immunized mice after restimulation with either the whole S-derived overlapping peptide pool (S) or with two individual S-derived CD8 T-cell epitopes, S_190_ and S_208_. It has been demonstrated that the specificity of murine S-specific CD8^+^ T-cell responses differs depending on whether they are primed by exogenous or endogenous HBsAg (31). Since epitope S_208_ is processed from exogenous but not endogenous HBsAg, and epitope S_190_ is processed from endogenous but not exogenous HBsAg (32). Distinguishing between these two specificities is of particular interest when comparing the immunogenicity of protein- and DNA-based vaccination.

**Figure 3:**
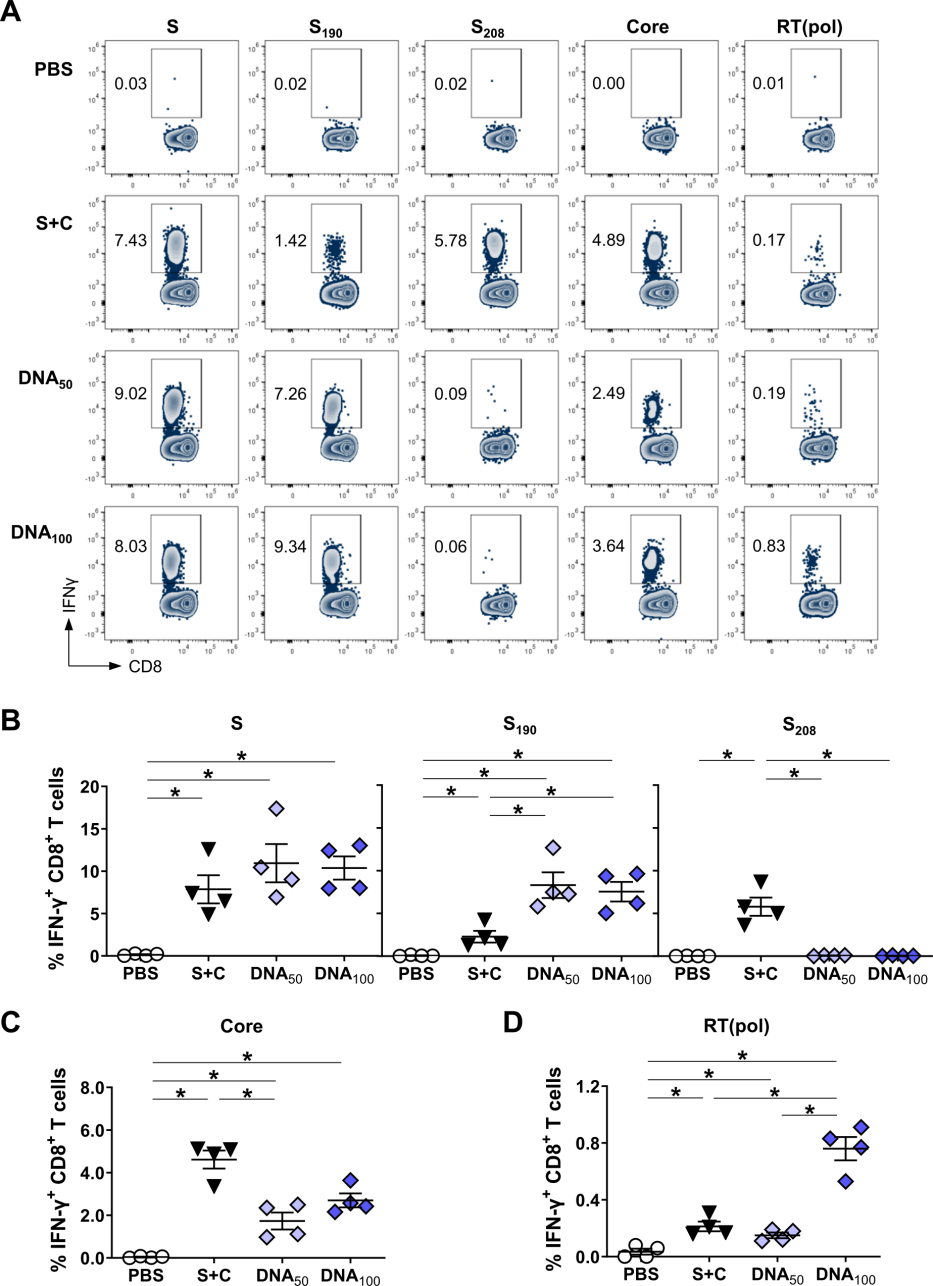
HBV-specific CD8^+^ T-cell responses induced by DNA prime – MVA boost immunization in HBV-naïve mice. C57BL/6J mice (groups n ≥ 4) were immunized as depicted in **Figure 2A**. End-point analyses were performed one week after MVA-HBVac boost, at week 5. **(A)** Representative flow cytometry plots of HBV-specific IFN-γ^+^ CD8^+^ T cells detected by intracellular cytokine staining (ICS) of splenocytes stimulated with HBV S-, Core-, and RT(pol)-specific overlapping peptide pools and with single peptides S_190_ and S_208_. **(B-D)** Frequencies of HBV S-, Core-, and RT(pol)-specific IFN-γ^+^ CD8^+^ T cells detected in splenocytes by ICS after *ex vivo* stimulation with corresponding peptide pools and single peptides S_190_ and S_208_. **(B-D)** Mean ± SEM is indicated. Statistical analysis was conducted using nonparametric One-Way ANOVA. *p*-values < 0.05 were considered statistically significant and marked with asterisks (*p < 0.05).

DNA- and protein-based vaccination regimens elicited robust and comparable S-specific IFN-γ CD8^+^ T-cell response in splenocytes stimulated with the S-derived peptide pool (**Figures 3A, B**). As expected, priming with DNA-HBVac resulted in significantly stronger S_190_-specific CD8^+^ T-cell responses than protein immunization, whereas mice primed with recombinant antigens showed mostly S_208_-specific CD8^+^ T-cell responses. The low S_190_-specific CD8^+^ T-cell response detected in the protein group might be attributed to the boosting effect of MVA, which delivers HBsAg in an endogenous manner. The Core-specific CD8^+^ T-cell response detected for DNA groups was stronger in mice receiving higher doses of DNA-HBVac (DNA_100_), but lower than the response detected for the protein group (**Figures 3A, C**). Moreover, priming with DNA-HBVac at 100 µg resulted in a vigorous RT(pol)-specific CD8^+^ T-cell response compared to the other examined regimens (**Figures 3A, D**).

Taken together, the vaccination regimen employing DNA-HBVac for priming elicited vigorous HBV-specific CD4^+^ and CD8^+^ T-cell responses, comparable in magnitude to those elicited by the recombinant proteins, but a poor anti-HBs response in HBV-naïve mice. Overall, only minor differences in immunogenicity were observed between the two tested DNA doses: 50 µg and 100 µg. Consequently, we selected both DNA doses to compare their efficacy in HBV carrier mice.

### 3.3 Priming with DNA-HBVac fails to elicit an effective anti-HBs response in HBV-carrier mice

To evaluate whether priming with DNA-HBVac could achieve immune control of persistent HBV infection, we analyzed the immunogenicity and antiviral efficacy of the DNA prime–MVA boost *TherVacB* regimen in HBV-carrier mice. Since mice are not naturally susceptible to HBV infection, we employed recombinant AAV to deliver 1.2-overlength HBV genome into the livers of immunocompetent C57BL/6J mice. This allows persistent HBV replication and protein expression in murine liver, with viral replication intermediates and transcripts detectable for at least 1 year after transduction. Moreover, the AAV-HBV mouse model is characterized by persistent HBsAg and HBeAg levels in the serum, no significant inflammation in the liver, and no HBV-specific antibody or T-cell responses (23).

**Figure 4:**
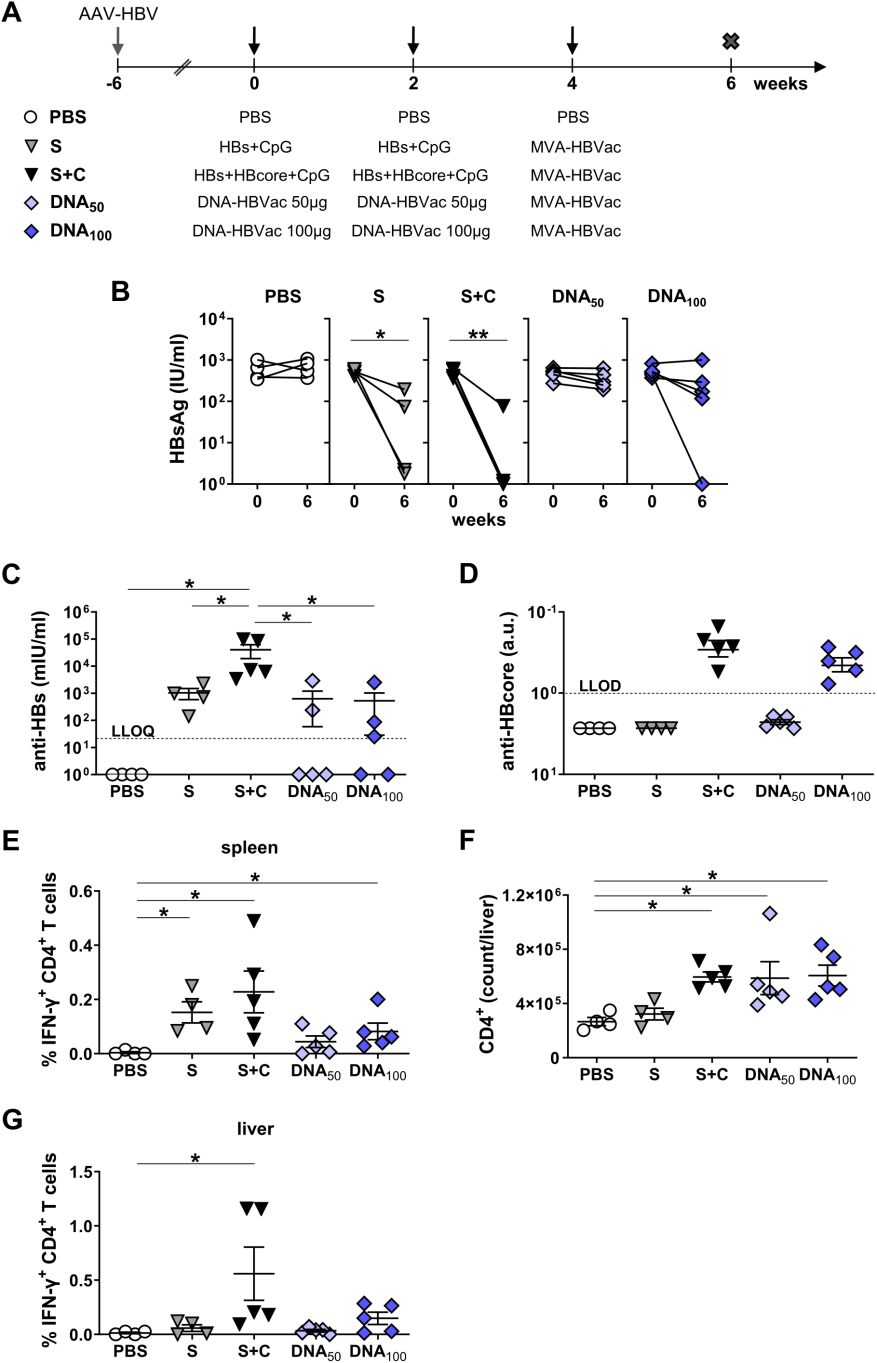
HBV-specific humoral and S-specific CD4^+^ T-cell responses induced by DNA prime – MVA boost immunization in HBV carrier mice. **(A)** C57BL/6J mice (groups *n* ≥ 4) were infected with AAV-HBV six weeks prior to the vaccination to establish persistent HBV replication. Starting at week 0, mice were immunized twice in a two-week interval with 10 µg of recombinant HBsAg adjuvanted with 15 µg CpG **(S)**, a mixture of adjuvanted 10µg each HBsAg and HBcoreAg (S+C), or DNA-HBVac at a 50 µg or 100 µg dose (DNA_50_ or DNA_100_, respectively). Boost with MVA-HBVac (3 × 10^7^ IFU/mouse) was administered two weeks later, at week 4. Mice receiving PBS injections served as the control group. Mice were analyzed at week 6, two weeks after the MVA boost. **(B)** Serum HBsAg levels were detected before the first vaccination (week 0) and two weeks after the last vaccination (week 6). Serum levels of **(C)** anti-HBs and **(D)** anti-HBcore antibodies at week 6. **(E)** Splenic IFN-γ^+^ CD4^+^ T cells after *ex vivo* stimulation with overlapping HBV S-specific peptide pool. **(F)** Numbers of CD4^+^ T cells in the livers of mice were determined by flow cytometry using CountBright™ absolute counting beads. **(G)** Liver-associated IFN-γ^+^ CD4^+^ T cells after *ex vivo* stimulation with overlapping HBV S-specific peptide pool. **(C-G)** Mean ± SEM is shown. Statistical analysis was conducted using nonparametric One-Way ANOVA. *p*-values < 0.05 were considered statistically significant and marked with asterisks (*p < 0.05, **p < 0.01, ns– not significant). a.u.- arbitrary units; LLOD– lower limit of detection; LLOQ– lower limit of quantification.

We thus infected C57BL/6J mice with AAV-HBV six weeks before the start of immunizations, allowing for the establishment of persistent HBV replication and solid immune tolerance to HBV antigens (8, 11). Mice received two priming immunizations with either 50 µg or 100 µg of DNA-HBVac (DNA_50_; DNA_100_, respectively) at weeks 0 and 2, followed by a booster immunization with MVA-HBVac at week 4 (**Figure 4A**). Endpoint analyses were performed two weeks after the boost, at week six. For comparison, *TherVacB* regimens employing either an adjuvanted mixture of recombinant HBsAg and HBcoreAg (S+C), or only adjuvanted HBsAg (S) for priming were used. HBV-carrier mice receiving PBS injections served as the control group.

We first determined the impact of *TherVacB* regimens on the reduction of serum HBsAg levels by monitoring the mice at the onset (week 0) and the endpoint (week 6) of the experiment. 2/4 mice vaccinated with recombinant HBsAg and even 4/5 mice vaccinated with the mixture of both recombinant proteins showed an impressive >3-log_10_ reduction in HBsAg levels at week 6 (**Figure 4B**). In contrast, priming with 50 µg of DNA-HBVac resulted in only a minor decrease in serum HBsAg levels. The 100 µg dose resulted in a <1-log_10_ reduction of HBsAg in 2/4 mice, and 1/5 mice became HBsAg-negative by week 6.

The decrease in serum HBsAg levels correlated with the amount of anti-HBs induced by immunization. The highest anti-HBs titers were detected in mice immunized with a mixture of adjuvanted protein antigens. In contrast, mice primed with DNA showed only low or undetectable anti-HBs levels (**Figure 4C**). Consistent with observations in HBV-naïve mice, these results indicate that priming with DNA-HBVac induces only low anti-HBs titers, insufficient to neutralize circulating HBsAg. The group of mice primed with the adjuvanted HBsAg only showed lower anti-HBs levels than the mice immunized with the mixture of HBs and HBcore antigens. Even though DNA priming failed to induce a strong anti-HBs response, anti-HBcore antibodies were detected in the DNA_100_ group at similar levels as in the S+C group (**Figure 4D**).

The levels of anti-HBs correlated with the magnitude of S-specific CD4^+^ T-cell response detected in the spleens of the immunized mice at week 6 (**Figure 4E**). Mice receiving priming immunization with the mixture of recombinant S and Core antigens or DNA-HBVac, but not with HBsAg alone, showed significantly higher numbers of CD4^+^ T cells in the liver than PBS controls (**Figure 4F**). High frequencies of IFN-γ^+^ S-specific CD4^+^ T cells, however, were detected in mice primed either with HBsAg alone or with the mixture of recombinant S and Core antigens, whereas most of the mice primed with DNA-HBVac showed very low S-specific IFN-γ CD4^+^ T-cell response. In liver-associated lymphocytes, strong IFN-γ S-specific CD4^+^ T-cell responses were only detected in 2/5 mice primed with the recombinant antigens. The remaining 3/5 mice showed weaker responses comparable to those primed with 100 µg of DNA-HBVac (**Figure 4G**). No Core- or RT(pol)-specific IFN-γ CD4^+^ T-cell responses could be detected in the livers and spleens of immunized HBV-carrier mice (**Supplementary Figures 1A, B**).

Taken together, these data demonstrate that immunization with high doses of DNA-HBVac can prime an HBV-specific CD4^+^ T-cell response and elicit high titers of anti-HBcore, but not anti-HBs antibodies. Compared with priming with adjuvanted proteins, however, it results in inefficient neutralizing anti-HBs induction, leading to poor HBsAg seroconversion.

### 3.4 Priming with DNA-HBVac induces a broad, effector virus-specific CD8^+^ T-cell response capable of eliminating hepatocytes that persistently replicate HBV

**Figure 5:**
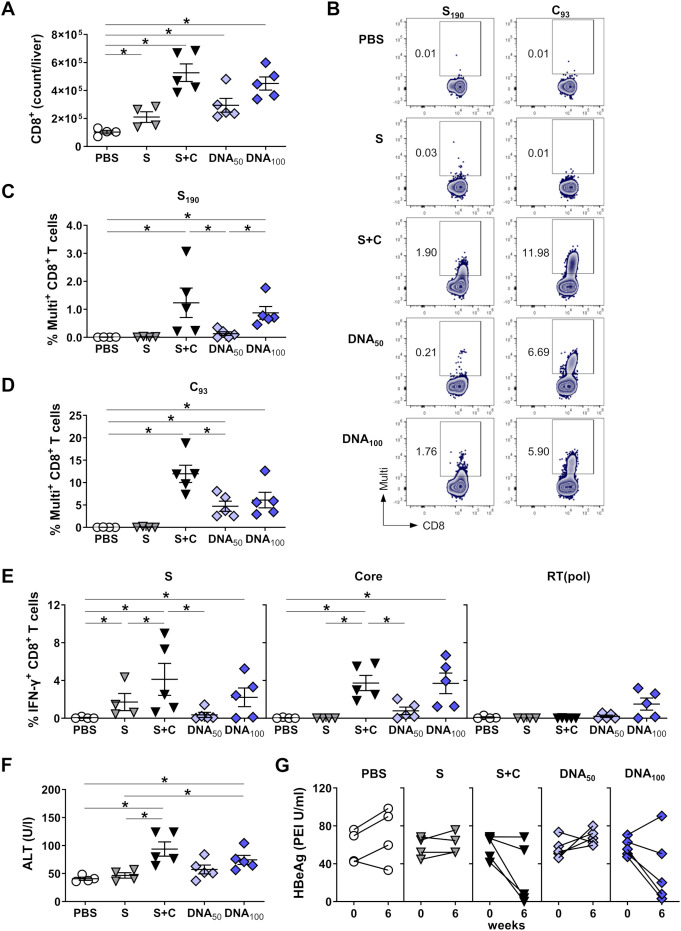
HBV-specific CD8^+^ T-cell responses induced by DNA prime – MVA boost immunization in HBV carrier mice. C57BL/6J mice were infected (groups *n* ≥ 4) with AAV-HBV and immunized as depicted in **Figure 4A**. End-point analyses were performed two weeks after MVA boost, at week 6. **(A)** Numbers of CD8^+^ T cells in the livers of mice were determined by flow cytometry using CountBright™ absolute counting beads. **(B)** Representative flow cytometry plots of S and Core-specific CD8^+^ T cells detected with S_190_- or C_93_-specific MHC-I in isolated liver-associated lymphocytes. **(C-D)** Frequencies of hepatic **(C)** S- and **(D)** Core-specific CD8 T cells detected with S_190_- or C_93_-specific MHC-I multimers. **(E)** HBV S-, Core- and RT(pol)-specific IFN-γ responses of hepatic CD8^+^ T cells determined by ICS after *ex vivo* stimulation with overlapping HBV S-, Core- and RT(pol)-specific peptide pools. **(F)** Serum ALT peak activity value detected for the individual mice between weeks 0 and 6. **(G)** Serum HBeAg levels detected before the first immunization (week 0) and two weeks after the last vaccination (week 6). **(A, C-F)** Mean ± SEM is shown. Statistical analysis was conducted using nonparametric One-Way ANOVA. *p*-values < 0.05 were considered statistically significant and marked with asterisks (*p < 0.05).

Since effector HBV-specific CD8^+^ T cells are crucial for eliminating HBV-infected hepatocytes, we next compared the magnitude and functionality of *TherVacB*-induced CD8^+^ T-cell responses in HBV-carrier mice primed with DNA-HBVac or recombinant proteins (shown in **Figure 4A**). The numbers of liver-associated CD8^+^ T cells in all immunized groups were significantly elevated compared with PBS-treated mice, especially in those primed with the mixture of recombinant S and Core antigens and a high dose of DNA-HBVac (**Figure 5A**). To detect HBV-specific CD8^+^ T cells, we performed direct *ex vivo* staining of isolated splenic and hepatic lymphocytes with MHC multimers loaded with S-derived peptide S_190_ and Core-derived peptide C_93_ two weeks after completing the vaccination scheme, at week 6. Unfortunately, as previously reported by others (33), we were unable to generate MHC-I multimers specific for the S_208_ peptide. The frequencies of hepatic S_190_-specific CD8^+^ T cells were high in mice receiving priming with 100 µg of DNA-HBVac compared to those primed with a mixture of recombinant proteins (**Figures 5B,C**). Notably, immunization with 50 µg of plasmid DNA induced barely any S_190_-specific CD8^+^ T cells in HBV-carrier mice. In the groups of mice primed with DNA, high frequencies of hepatic C_93_-specific CD8^+^ T cells could be detected, irrespective of the administered dose, albeit at lower levels than in mice immunized with a mixture of adjuvanted HBsAg and HBcoreAg VLPs (**Figures 5B, D**). Although immunization with DNA and a mixture of recombinant antigens resulted in high frequencies of antigen-specific CD8^+^ T cells in the livers of HBV-carrier mice, barely any S_190_- and C_93_-specific CD8^+^ T cells were detected in the spleens (**Supplementary Figures 2A, B**), indicating that two weeks after the boost, the vaccine-elicited CD8^+^ T cells remain mainly in the liver, at the site of the infection.

To examine whether vaccine-elicited HBV-specific CD8^+^ T-cells are also functional, we analyzed the secretion of IFN-γ by ICS. Priming strategies relying on adjuvanted recombinant proteins and high doses of DNA-HBVac elicited vigorous S-specific IFN-γ CD8^+^ T-cell responses in the livers of mice (**Figure 5E**). In the spleens, low S_208_-specific CD8^+^ T-cell responses could be detected in mice primed with HBsAg alone or with a mixture of recombinant proteins, whereas no S_190_-specific CD8^+^ T-cell response was observed for any vaccination regimen (**Supplementary Figure 2C**). Priming immunization with 100 µg of DNA-HBVac elicited a comparably strong Core-specific IFN-γ CD8^+^ T-cell response in the liver, as the mixture of recombinant HBsAg and HBcoreAg. Only in the DNA_100_ group could we detect a significant hepatic RT(pol)-specific IFN-γ CD8^+^ T-cell response in 3 out of 5 mice (**Figure 5E**). By contrast, in mice primed with a lower dose of DNA-HBVac, functional S-, Core-, and RT(pol)-specific IFN-γ^+^ CD8^+^ T cells were either weak or absent in the liver (**Figure 5E**) as well as in the spleen (**Supplementary Figures 2D, E**).

Consistently, with the strong multi-specific effector CD8^+^ T-cell response detected in HBV-carrier mice primed either with 100 µg of DNA-HBVac or with a mixture of recombinant proteins, substantial ALT elevations were observed only in these two groups of mice (**Figure 5F**). This suggests that, in addition to cytokine secretion, virus-specific CD8^+^ T cells elicited by the DNA prime-MVA boost regimen also exhibit cytolytic functions in the liver.

To further investigate the effect of priming with DNA-HBVac on reducing HBV replication, we monitored serum HBeAg levels at the onset and endpoint of the experiment and quantified HBcore^+^ hepatocytes two weeks after complete vaccination. Mice primed with only recombinant HBsAg or 50 µg of DNA-HBVac showed minor effects on HBeAg levels (**Figure 5G**). Comparable to what we have observed with a protein-based prime combining HBsAg and HBcoreAg VLPs, 3/5 mice that received a high priming dose of 100 µg DNA exhibited a profound decrease in serum HBeAg levels.

**Figure 6:**
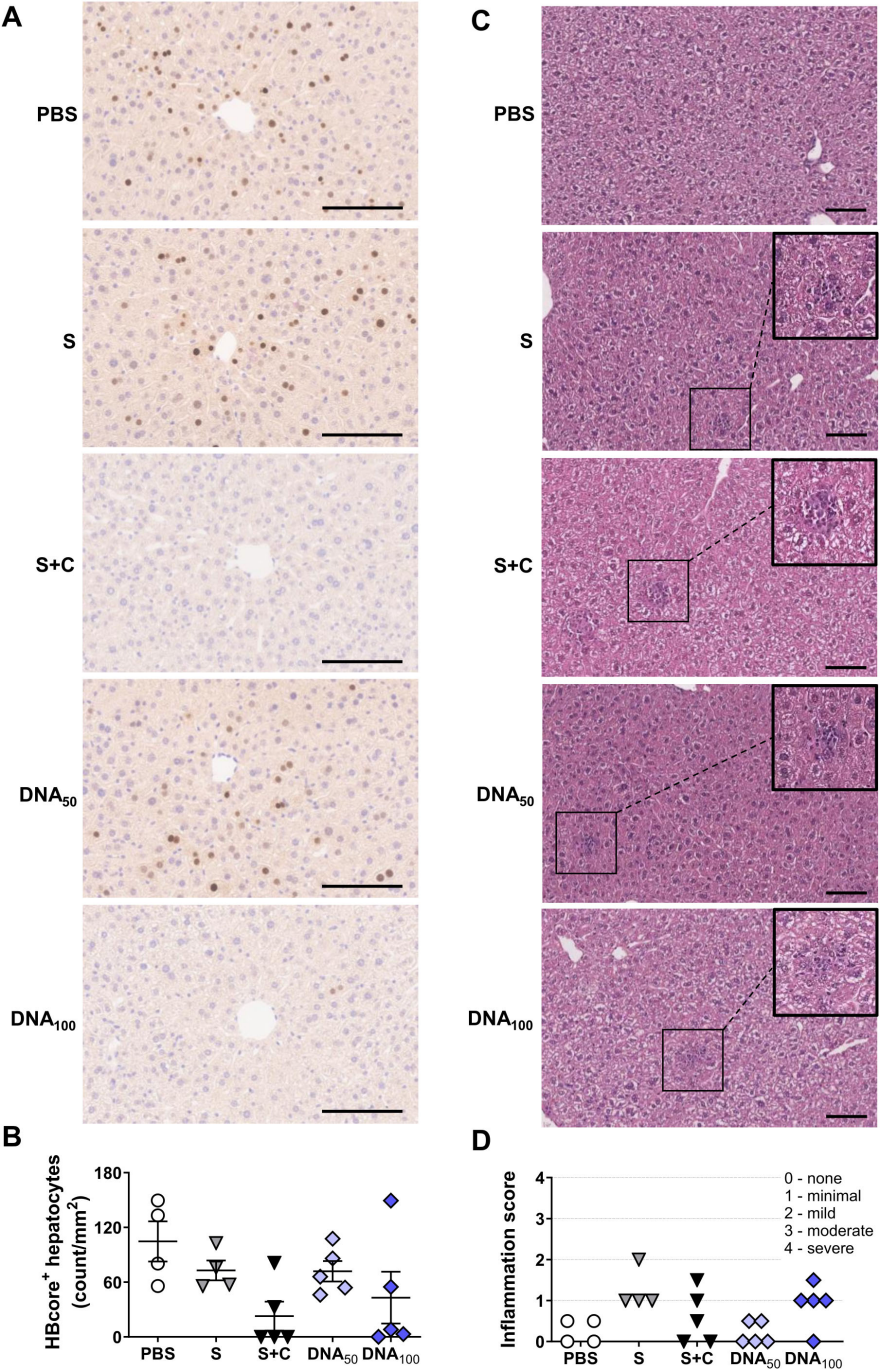
Antiviral and histopathological effects induced by DNA prime – MVA boost immunization in the livers of HBV carrier mice. C57BL/6J mice were infected (groups *n* ≥ 4) with AAV-HBV and immunized as depicted in **Figure 4A**. End-point analyses were performed two weeks after MVA boost, at week 6. **(A)** Representative images of liver immunohistochemistry staining for HBcore protein (brown). Scale bars represent 100 μm. **(B)** Quantification of the numbers of HBcore-positive hepatocytes per mm^2^. **(C)** Representative images of liver sections stained with H&E. Scale bars represent 100 μm, and inlets show magnification of the indicated areas. **(D)** Histological scoring to assess liver inflammation in individual mice. **(B)** Mean ± SEM is shown.

To substantiate our findings, we quantified the number of HBV-positive hepatocytes in the liver by immunohistochemistry two weeks after complete vaccination. The number of HBV^+^ hepatocytes in mice primed with only recombinant HBsAg or with 50 µg of DNA-HBVac remained high, while in three mice from S+C group barely any positive cells could be detected (**Figures 6A, B**). The three mice primed with a high DNA-HBVac dose, which significantly reduced HBeAg, eliminated over 95% of infected hepatocytes, with one mouse completely resolving persistent HBV infection by week 6 after the MVA boost. Thus, the antiviral effect of the vaccination observed in the DNA_100_ group was comparable to that of the protein prime-MVA boost regimen. Although immune cell infiltration into the liver parenchyme was observed in immunized mice independent of the vaccination regimen (**Figure 6C**), no adverse histopathological effects were observed, with at most a mild inflammation score (**Figure 6D**).

Taken together, these data demonstrate that priming with a high dose of DNA-HBVac can break HBV-specific CD8^+^ T cell tolerance, suppress HBV replication, and eliminate infected hepatocytes as efficiently as priming with the adjuvanted recombinant proteins, but induces only low neutralizing antibody titers. In addition, immunization with DNA broadened *TherVacB*-elicited immune responses by priming RT(pol)-specific T cells, implying that the poor induction of neutralizing anti-HBs is the major obstacle for DNA-HBVac priming.

### 3.5 Simultaneous priming with DNA-HBVac and recombinant HBsAg inhibits plasmid-mediated CD8^+^ T-cell immunity

To enhance the anti-HBs response elicited by DNA immunization, we investigated whether simultaneous priming with DNA-HBVac and recombinant HBsAg would increase S-specific B-cell responses and, consequently, enhance reductions in circulating HBsAg. We also wondered whether such a combination would require the addition of a CpG adjuvant since plasmid DNA already contains intrinsic CpG motifs (34) that could provide sufficient immune stimulation, and whether an additional adjuvant could enhance the immunogenicity of our DNA vaccine, as previously reported (35).

**Figure 7:**
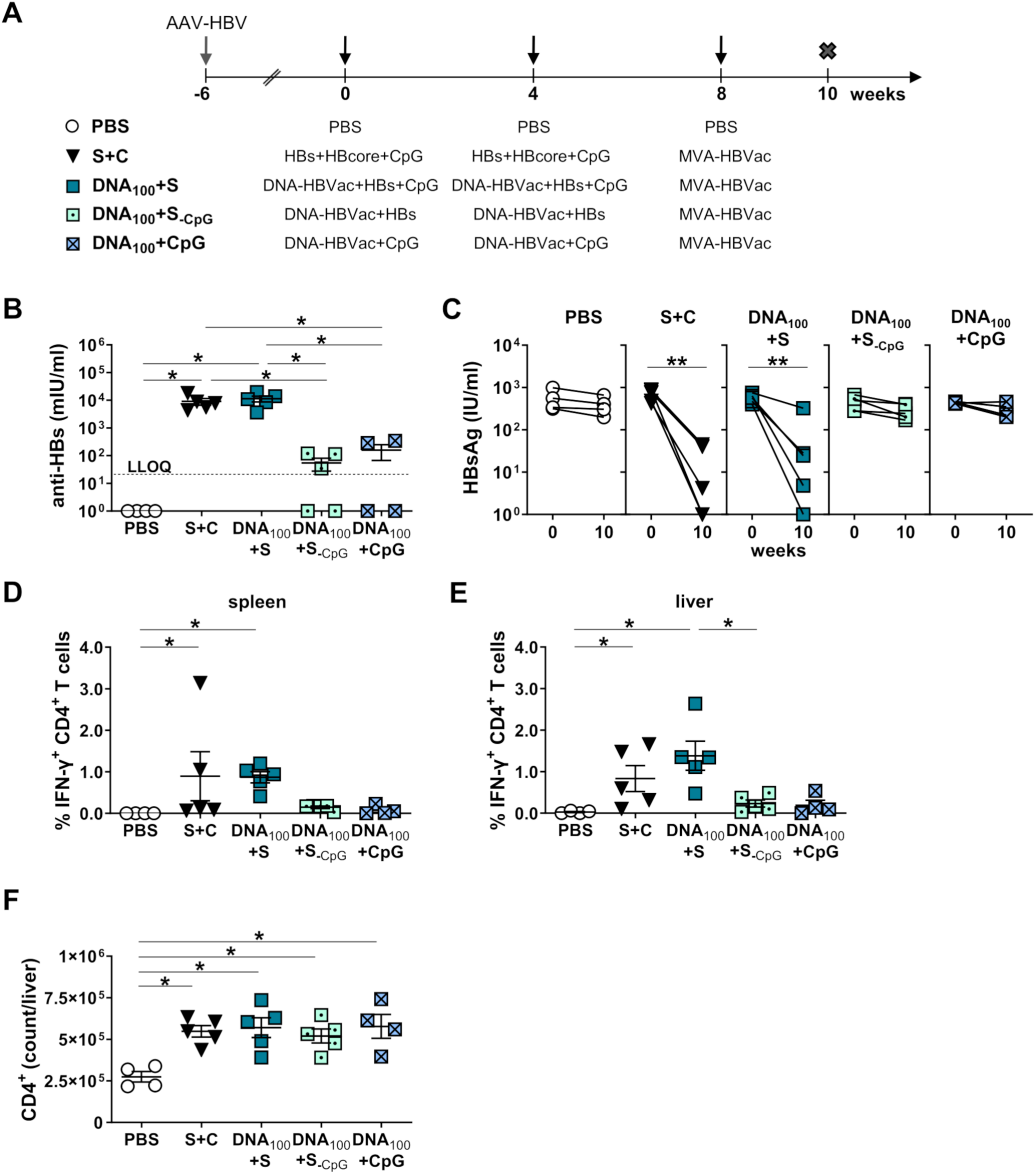
HBV-specific humoral and S-specific CD4^+^ T-cell responses induced by simultaneous DNA/HBsAg prime – MVA boost regimen in HBV-carrier mice. **(A)** C57BL/6J mice (groups *n* ≥ 4) were infected with AAV-HBV six weeks prior to the vaccination to establish persistent HBV replication. Starting at week 0, mice were immunized twice in a four-week interval with a mixture of adjuvanted 10 µg each recombinant HBsAg and HBcoreAg (S+C), a mixture of adjuvanted recombinant HBsAg and DNA-HBVac at 100 µg dose (DNA_100_+S), a mixture of DNA-HBVac at 100 µg dose and recombinant particulate HBsAg without adjuvant (DNA_100_+S_-CpG_), or DNA-HBVac at 100 µg dose adjuvanted with 15 µg CpG (DNA_100+CpG_). Boost with MVA-HBVac (3 × 10^7^ IFU/mouse) was administered at week 10. Mice receiving PBS served as controls (PBS). Mice were analyzed at week 10, two weeks after the MVA boost. Serum levels of **(B)** anti-HBs antibodies at week 10. **(C)** Serum HBsAg levels detected before the first immunization (week 0) and two weeks after the last vaccination (week 10). **(D-E)** IFN-γ^+^ CD4^+^ T cells after *ex vivo* stimulation with overlapping HBV S-specific peptide pool isolated from **(D)** spleens and **(E)** livers of immunized mice. **(F)** Numbers of CD4^+^ T cells in the livers of mice were determined by flow cytometry using CountBright™ absolute counting beads. **(B, D-F)** Mean ± SEM is shown. Statistical analysis was conducted using nonparametric One-Way ANOVA. *p*-values < 0.05 were considered statistically significant and marked with asterisks (*p < 0.05, **p < 0.001). LLOQ– lower limit of quantification.

We infected the C57BL/6J mice with AAV-HBV and initiated immunizations six weeks later. In this experiment, we adjusted the immunization schedule to 4-week intervals, following the regimen used in our clinical trial. Mice were primed twice at weeks 0 and 4 with a mixture of 100 µg DNA-HBVac and recombinant HBsAg, either with or without CpG adjuvant (DNA_100_+S and DNA_100_+S_-CpG_, respectively), or the DNA only with CpG (DNA_100_+CpG). At week 8, mice were boosted with MVA-HBVac (**Figure 7A**). For comparison, mice received the standard *TherVacB* scheme with adjuvanted recombinant antigens (S+C) for priming or PBS as a negative control. We analyzed HBV-specific antibody and T-cell responses two weeks after the MVA boost, at week 10.

Simultaneous priming with DNA-HBVac and adjuvanted HBsAg induced comparably strong anti-HBs responses with mean anti-HBs levels of 10^4^ mIU/ml, as adjuvanted recombinant antigens (**Figure 7B**). However, priming the mice with a mixture of DNA and HBsAg without the CpG adjuvant (DNA_100_+S_-CpG_), resulted in very low anti-HBs titers. This indicates that, HBsAg without adjuvant doesn’t elicit an effective anti-HBs response. Following robust anti-HBs induction in the DNA_100_+S group, HBsAg levels dropped sharply from weeks 0 to 10 (**Figure 7C**). In contrast, priming with a mixture of DNA and HBsAg without adjuvant or adjuvanted DNA-HBVac did not result in any significant serum HBsAg reductions. Furthermore, the addition of adjuvanted HBsAg to the DNA vaccine significantly improved splenic and hepatic S-specific IFN-γ CD4^+^ T-cell responses, reaching levels similar to or even slightly higher than those in the S+C group (**Figures 7D, E**). In mice primed with DNA and HBsAg without adjuvant or with adjuvanted DNA-HBVac, only weak S-specific CD4^+^ T-cell responses were detected in the liver (**Figure 7E**), although the immunizations resulted in comparably high numbers of liver-associated CD4^+^ T cells as in S+C and DNA_100_+S groups (**Figure 7F**). None of the immunized mice demonstrated any IFN-γ Core- or RT(pol)-specific CD4^+^ T-cell responses at week 10 (*data not shown*).

**Figure 8:**
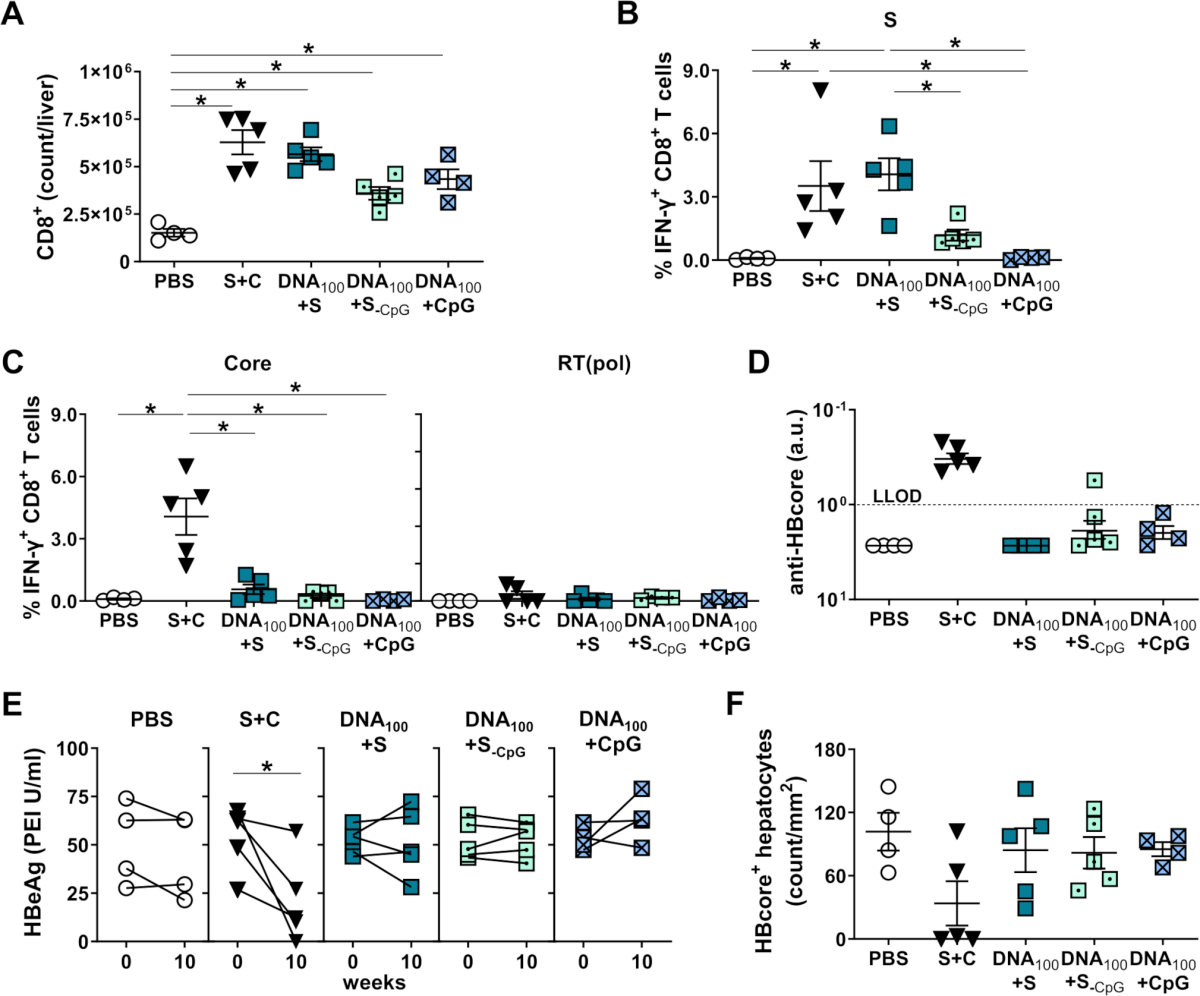
HBV-specific CD8^+^ T-cell responses induced by simultaneous DNA/HBsAg prime – MVA boost regimen in HBV-carrier mice. C57BL/6J mice were infected (groups *n* ≥ 4) with AAV-HBV and immunized as depicted in **Figure 7A**. End-point analyses were performed two weeks after MVA boost, at week 10. **(A)** Numbers of CD8^+^ T cells in the livers of mice were determined by flow cytometry using CountBright™ absolute counting beads. **(B)** S-specific and **(C)** Core- and RT(pol)-specific IFN-γ responses of hepatic CD8^+^ T cells determined after *ex vivo* stimulation with overlapping S-, Core- and RT(pol)-specific peptide pools. **(D)** Serum levels of anti-HBcore. **(E)** Serum HBeAg levels detected before the first immunization (week 0) and two weeks after the last vaccination (week 10). **(F)** Quantification of the numbers of HBcore-positive hepatocytes per mm^2^. **(A-D, F)** Mean ± SEM is shown. Statistical analysis was conducted using nonparametric One-Way ANOVA. *p*-values < 0.05 were considered statistically significant and marked with asterisks (*p < 0.05, **p < 0.001). a.u.- arbitrary units; LLOD– lower limit of detection.

We next quantified the numbers of liver-associated CD8^+^ T cells two weeks after booster immunization, at week 6. All vaccination regimens resulted in significantly higher numbers of CD8^+^ T cells in the livers than in PBS-treated mice, with the highest values observed for mice primed with the mixture of adjuvanted antigens or with the combination of DNA and adjuvanted HBsAg (**Figure 8A**). We detected a strong vaccine-elicited S-specific IFN-γ CD8^+^ T-cell response after stimulation of LALs with an S-derived overlapping peptide pool after priming with the combination of DNA and adjuvanted HBsAg (**Figure 8B**). Not only in the livers but also in the spleens, the frequencies of S-specific IFN-γ CD8^+^ T cells were comparable to the group of mice receiving a mixture of adjuvanted recombinant HBs and HBcore antigens (**Supplementary Figure 3A**). The frequencies of hepatic CD8^+^ T cells specific for endogenously processed epitope S_190_ in the DNA_100_+S group, however, were unexpectedly low for DNA immunization and were not different than those in the mice primed with the S+C regimen (**Supplementary Figure 3B**). This indicated that the S-specific CD8^+^ T-cell response was predominantly primed by adjuvanted recombinant HBsAg rather than by S or L proteins expressed by the DNA vaccine. Unexpectedly, simultaneous priming with a mixture of DNA and adjuvanted HBsAg failed to induce any hepatic Core-specific CD8^+^ T-cells (**Supplementary Figure 3C**). No functional Core- and RT(pol)-specific IFNγ CD8^+^ T-cell responses were observed in the livers and the spleens of immunized mice (**Figure 8C**; **Supplementary Figure 3D**).

A Core-specific CD8^+^ T-cell response was detected upon priming with a mixture of recombinant HBs and HBcore antigens, which was significantly more immunogenic than the other vaccination regimens. Notably, anti-HBcore antibodies were also not detected in mice simultaneously primed with DNA-HBVac and adjuvanted HBsAg (**Figure 8D**), suggesting the ‘silencing’ of immune responses toward the antigens expressed by the DNA vaccine in the presence of adjuvanted protein.

The strength of the effector hepatic CD8^+^ T-cell response correlated with reductions in serum HBeAg levels and the number of HBV^+^ cells in the liver. All mice primed with a mixture of adjuvanted recombinant antigens demonstrated a considerable reduction in serum HBeAg, and in 3/5 mice, barely any HBcore^+^ hepatocytes were detected in the liver at the end of the experiment (**Figures 8E, F**). HBeAg remained constant in mice immunized with a combination of DNA-HBVac and adjuvanted HBsAg (**Figure 8E**). Only two mice in the DNA_100_+S group experienced a modest reduction in HBeAg levels after vaccination, and the number of infected hepatocytes remained comparable to that of the PBS controls (**Figure 5I**). Priming with DNA and HBsAg without adjuvant or with adjuvanted DNA-HBVac did not affect serum HBeAg nor the numbers of HBV^+^ hepatocytes (**Figures 8E, F**).

These results demonstrate that simultaneous priming immunization with DNA and adjuvanted recombinant HBsAg improves anti-HBs titers and induces HBsAg loss, thereby achieving efficacy comparable to that of immunization with a mixture of adjuvanted recombinant HBV antigens. The low immunogenicity of the DNA_100_+S_-CpG_ and DNA_100_+CpG regimens demonstrated that plasmid DNA cannot substitute for the CpG adjuvant, and that the addition of CpG cannot enhance the efficacy of DNA vaccination. In the presence of HBsAg, DNA immunization failed to induce proper HBV-specific CD8^+^ T-cell responses, resulting in a minor effect on HBeAg levels and the number of HBV-positive hepatocytes. The lack of CD8^+^ T cells directed against Core and RT(pol) antigens, which represent important effectors of HBV clearance, indicates that simultaneous priming with DNA and HBsAg preferentially skews the vaccine-induced immune responses toward the recombinant antigen but does not elicit an immune response to the antigens expressed by DNA-HBVac.

### 3.6 Sequential priming with DNA-HBVac and recombinant HBsAg induces robust HBV-specific immune responses in HBV-carrier mice

**Figure 9:**
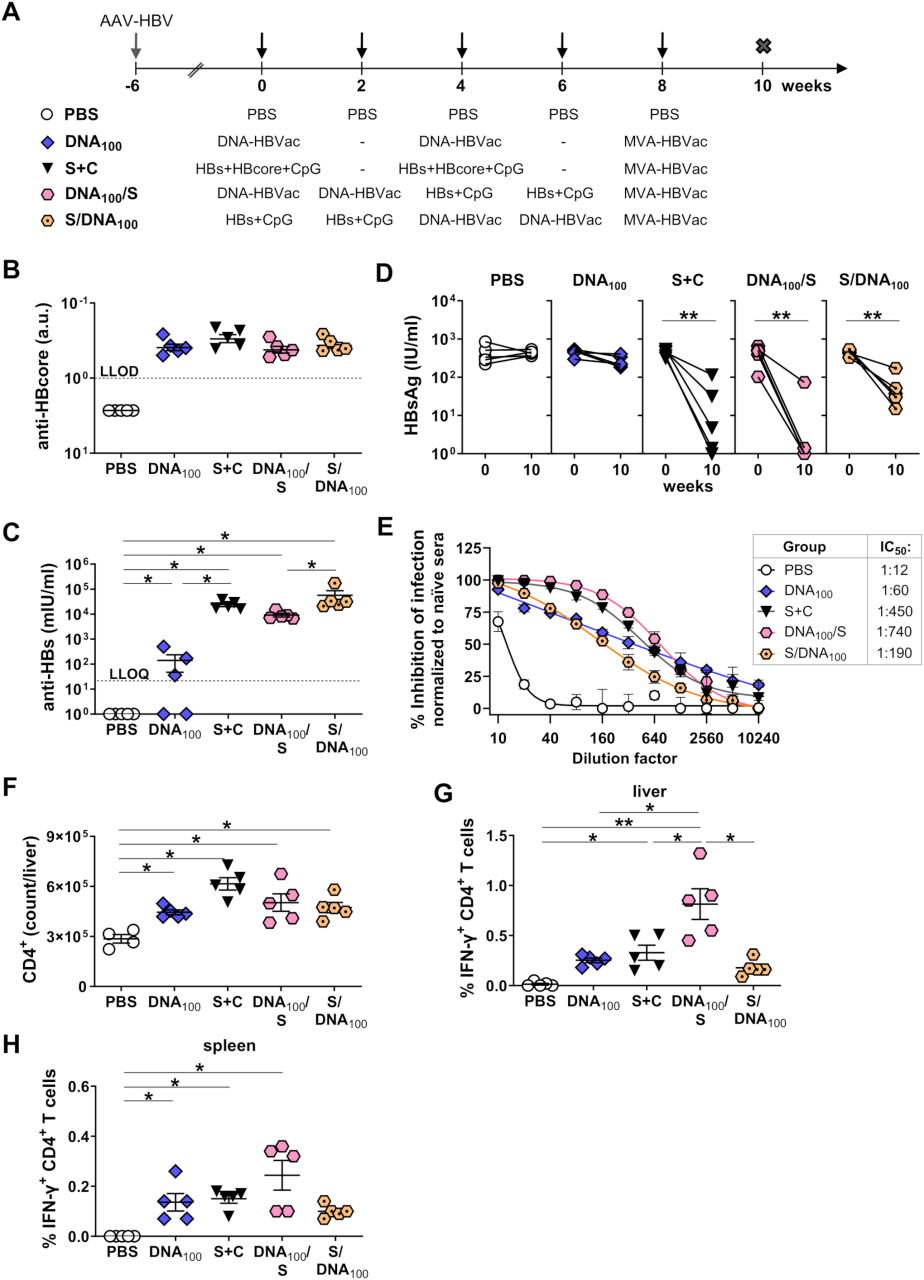
HBV-specific humoral and S-specific CD4^+^ T-cell responses induced by sequential DNA and HBsAg prime – MVA boost immunization in HBV-carrier mice. **(A)** C57BL/6J mice (groups *n* = 5) were infected with AAV-HBV six weeks prior to the vaccination to establish persistent HBV replication. Starting at week 0 mice were sequentially immunized four times in a two-week interval with twice DNA-HBVac at 100 µg dose followed by two immunizations with adjuvanted recombinant HBsAg (DNA_100_/S) or twice with adjuvanted HBsAg followed by two immunizations with DNA-HBVac at 100 µg dose (S/DNA_100_). For reference mice primed twice in a four-week interval with a mixture of adjuvanted 10µg each recombinant HBsAg and HBcoreAg (S+C) or with DNA-HBVac at 100 µg dose (DNA_100_) were used. Boost with MVA-HBVac (3 × 10^7^ IFU/mouse) was administered at week 8. Mice receiving PBS served as controls (PBS). Mice were analyzed at week 10, two weeks after the MVA boost. Serum levels of **(B)** anti-HBcore and **(C)** anti-HBs antibodies at week 10. **(D)** Serum HBsAg levels detected before the first immunization (week 0) and two weeks after the last vaccination (week 10). **(E)** The percent inhibition of infection for all immunization regimens at a range of pooled sera dilutions at week 10 was determined by a neutralization assay using recombinant HBV expressing NanoLuc. IC_50_ values determined from neutralization curves are shown. **(F)** Numbers of CD4^+^ T cells in the livers of mice were determined by flow cytometry using CountBright™ absolute counting beads. **(G-H)** IFN-γ^+^ CD4^+^ T cells after *ex vivo* stimulation with overlapping HBV S-specific peptide pool isolated from **(G)** livers and **(H)** spleens of immunized mice. (B, C; E-H) Mean ± SEM is shown. Statistical analysis using nonparametric One-Way ANOVA. *p*-values < 0.05 were considered statistically significant and marked with asterisks (*p < 0.05, **p < 0.001). a.u.- arbitrary units; LLOD– lower limit of detection; LLOQ– lower limit of quantification, IC_50_- half-maximal inhibitory concentration of infectivity.

Since simultaneous priming with DNA and recombinant HBsAg entirely abolished plasmid-elicited immunity, we next investigated whether sequential vaccinations with DNA and recombinant HBsAg could simultaneously induce HBV-specific humoral and cellular immune responses in mice persistently replicating HBV. We infected C57BL/6J mice with AAV-HBV and vaccinated them sequentially twice with 100 µg DNA-HBVac and twice with adjuvanted HBsAg at two-week intervals, starting the immunizations either with the DNA vaccine (DNA_100_/S) or with the recombinant antigen (S/DNA_100_). For reference, priming regimens consisting of two immunizations, administered four weeks apart, were used with either a mixture of adjuvanted recombinant HBsAg and HBcoreAg (S+C) or 100 µg of DNA-HBVac (DNA_100_). Two weeks after the last priming immunization (week 8), all groups of mice were boosted with MVA-HBVac. Mice receiving PBS injections (PBS) served as controls. We analyzed vaccine-elicited HBV-specific immune responses two weeks after the MVA boost, at week 10 (**Figure 9A**).

Analysis of anti-core antibody levels in the sera of mice at week 10 demonstrated that sequential priming with DNA-HBVac and adjuvanted HBsAg, independent of the order of the immunizations, elicited a significant core-specific antibody response, which was comparable to priming either with DNA only or with a mixture of adjuvanted HBsAg and HBcoreAg (**Figure 9B**). Importantly, both sequential regimens considerably improved anti-HBs responses as compared to priming only with DNA-HBVac (**Figure 9C**), especially if the immunizations began with adjuvanted recombinant HBsAg (S/DNA_100_).

Following induction of robust anti-HBs response, a significant decline in serum HBsAg levels was observed in all immunization groups using adjuvanted HBsAg for priming (S+C, DNA_100_/S, and S/DNA_100_), but not in mice primed only with DNA (DNA_100_) (**Figure 9D**). Sequential priming starting with DNA-HBVac (DNA_100_/S) resulted in the strongest decrease in serum HBsAg levels with a 3-log_10_ reduction in HBsAg levels measured at week 10 in 4/5 mice compared to the baseline values at week 0. Interestingly, sequential priming, starting with adjuvanted HBsAg, resulted in a less pronounced decrease in serum HBsAg (S/DNA_100_), despite the group showing the highest anti-HBs levels.

To understand the reason for this discrepancy, we analyzed the neutralization capacity of the sera against infection with a recombinant HBV expressing NanoLuc (rHBV) in HepG2-NTCP cells, and determined the half-maximal inhibitory concentration of infectivity (IC_50_) in serially diluted pooled samples collected at week 10. We found that sera of mice receiving sequential priming starting with DNA-HBVac (DNA_100_/S) exhibited the highest rHBV neutralization activity with an IC_50_ value corresponding to a 1:760 dilution (**Figure 9E**). By contrast, sera of mice primed first with adjuvanted HBsAg (S/DNA_100_) showed 4-fold reduced rHBV neutralization activity (IC_50_ ≈ 1:190 dilution), which was even lower than values detected for mice receiving only two priming immunizations with a mixture of recombinant antigens (S+C; IC_50_ ≈ 1:450 dilution). Since the assay can only determine the neutralizing activity of anti-HBs not complexed with HBsAg, these results suggest that mice receiving a sequential regimen starting with DNA-HBVac (DNA_100_/S) could neutralize HBsAg more effectively than the reverse priming regimen.

All vaccination regimens resulted in elevated numbers of CD4^+^ T cells in the liver (**Figure 9F**), but it was the magnitude of hepatic vaccine-elicited S-specific IFN-γ CD4^+^ T-cell response, which correlated with the extent of serum HBsAg reduction by the sequential priming. Initiating *TherVacB* vaccinations with DNA resulted in a superior hepatic S-specific IFN-γ CD4^+^ T-cell response (**Figure 9G**) compared to the sequential priming, which began with the adjuvanted HBsAg and showed a similar trend in splenocytes (**Figure 9H**). Neither of the vaccination protocols elicited significant Core- and RT(pol)-specific IFN-γ CD4^+^ T-cell responses in the livers or the spleens of immunized HBV-carrier mice (**Supplementary Figures 4A, B**).

**Figure 10:**
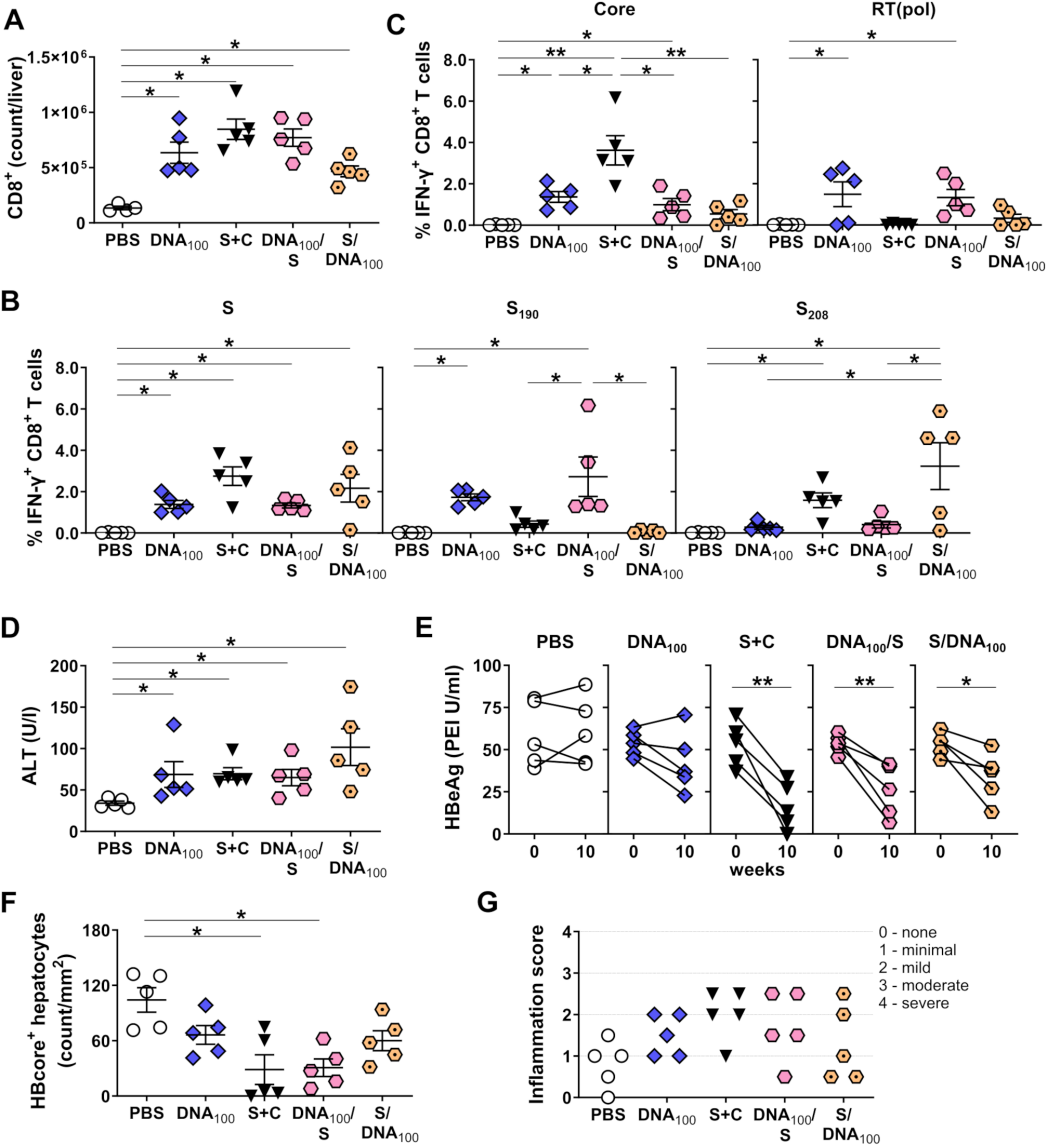
HBV-specific CD8^+^ T-cell responses induced by sequential DNA and HBsAg prime – MVA boost immunization in HBV-carrier mice. C57BL/6J mice (groups *n* = 5) were infected with AAV-HBV and immunized as depicted in **Figure 9A**. End-point analyses were performed two weeks after MVA boost, at week 10. **(A)** Numbers of CD8^+^ T cells in the livers of mice were determined by flow cytometry using CountBright™ absolute counting beads. **(B, C)** Frequencies of hepatic S-, Core- and RT(pol)-specific IFN-γ^+^ CD8^+^ T cells detected by ICS after *ex vivo* stimulation with HBV S-, Core-, RT(pol)-specific overlapping peptide pools and with single peptides S_190_ and S_208_. **(D)** Serum ALT peak activity value detected for the individual mice between week 0 and 10. **(E)** Serum HBeAg levels detected before the first immunization (week 0) and two weeks after the last vaccination (week 10). **(F)** Quantification of the numbers of HBcore-positive hepatocytes per mm^2^. **(G)** Histological scoring to assess liver inflammation in individual mice. Scale bars represent 100 μm. **(A-D, F)** Mean ± SEM is shown. Statistical analysis using nonparametric One-Way ANOVA. *p*-values < 0.05 were considered statistically significant and marked with asterisks (*p < 0.05, **p < 0.01).

All groups of vaccinated mice demonstrated increased numbers of CD8^+^ T cells in the liver (**Figure 10A**) and elicited vigorous hepatic S-specific IFN-γ CD8^+^ T cell responses (**Figure 10B**). Notably, sequential immunization, starting with DNA-HBVac, skewed the S-specific IFN-γ CD8^+^ T-cell response toward the endogenously presented epitope S_190_. In contrast, initiation of vaccinations with adjuvanted recombinant HBsAg resulted in S_208_-reactive CD8^+^ T cells in the liver (**Figure 10B**) and spleen (**Supplementary Figure 5A**). Consequently, S_208_-specific CD8^+^ T cells could not be detected in most mice receiving the DNA_100_/S priming regimen, and no S_190_-specific CD8^+^ T cells could be detected in mice receiving S/DNA_100_ immunizations. In contrast to simultaneous priming with DNA-HBVac and HBsAg, sequential DNA immunization followed by adjuvanted HBsAg and MVA-HBVac elicited vigorous Core- and RT(pol)-specific IFN-γ^+^ CD8^+^ T-cell response in the liver (**Figure 10C**), and in the spleen (**Supplementary Figure 5B**). Of note, in the group of mice sequentially vaccinated first with adjuvanted HBsAg, then with DNA-HBVac and MVA-HBVac, only very weak Core- and RT(pol)-specific CD8^+^ T-cell responses could be detected at the examined time-point. This indicates that the DNA_100_/S priming regimen is overall more immunogenic than S/DNA_100_ in HBV-carrier mice. Nevertheless, most immunized mice exhibited significant ALT elevations throughout the experiment (**Figure 10D**). Thus, significant reductions in serum HBeAg levels were observed for both sequential priming regimens and the *TherVacB*, which included a mixture of adjuvanted recombinant HBsAg and HBcoreAg (**Figure 10E**). However, sequential DNA immunization followed by adjuvanted HBsAg reduced the numbers of HBV-infected hepatocytes, on average, by 70% compared to PBS controls, whereas the reverse regimen reduced them by only 40% (**Figure 10F**; representative immunohistochemistry images in **Supplementary Figure 6A**). This indicates that the DNA_100_/S priming is overall more effective than S/DNA_100_ in HBV-carrier mice. In addition, neither vaccination regimen resulted in histopathological changes (**Supplementary Figure 6B**), or severe inflammation (**Figure 10G**).

In contrast to the initial experiments, mice receiving priming vaccinations only with DNA-HBVac (DNA100) showed no or only a modest drop in HBeAg levels (**Figure 10E**), and only a minor reduction in the number of infected hepatocytes (**Figure 10F**). This implies that DNA vaccination is more effective when administered at 2-week intervals, rather than 4-week intervals.

Taken together, these results demonstrate that sequential priming with DNA-HBVac and adjuvanted recombinant HBsAg can successfully elicit strong HBV-specific cellular immunity and anti-HBs responses, capable of eliminating HBV from the serum and liver in carrier mice. However, initiating vaccinations with DNA, followed by adjuvanted HBsAg and MVA-HBVac, induced high anti-HBs titers and more functional virus-specific CD4^+^ and CD8^+^ T-cell responses than the reverse regimen, thereby resulting in overall better antiviral effects. Thus, it proves a more suitable priming regimen for therapeutic vaccination. Although the priming regimen was extended to four immunizations, it did not overall improve the immunogenicity or efficacy of *TherVacB* compared with the classical regimen, which employs two injections of adjuvanted recombinant HBsAg and HBcoreAg.

## 4 Discussion

Developing an effective therapeutic hepatitis B vaccine remains challenging, as it requires concomitantly eliciting potent antibody, CD4^+^, and CD8^+^ effector T-cell responses in the presence of strong HBV-specific immune tolerance. Heterologous prime-boost regimens are currently the most efficient vaccination approaches against chronic diseases and cancer, as they allow for the stimulation of various branches of the immune system by combining distinct vaccine platforms (6, 12, 36–38). In this study, we investigated the immunogenicity of a therapeutic hepatitis B vaccine that employs priming with plasmid DNA and a boost with a recombinant MVA vector expressing a matched set of five HBV proteins optimized to induce a broad immune response against the most prevalent HBV genotypes. Our study demonstrates that DNA prime induced robust HBV-specific CD8^+^ T cell responses directed against multiple antigens, suggesting a potential for a broadly applicable therapeutic vaccine; however, it elicited poor anti-HBs titers and failed to efficiently reduce circulating HBsAg levels in mice persistently replicating HBV. To enhance the anti-HBs response, we combined DNA with adjuvanted HBsAg for priming, as strong anti-HBs induction and profound HBsAg loss were observed upon immunization with the established *TherVacB* regimen, which relies on priming with recombinant antigens. We demonstrate that sequential, but not simultaneous, immunizations initiated with DNA followed by recombinant HBsAg and MVA significantly enhance anti-HBs responses and support broad DNA-mediated CD8+ T-cell responses, ultimately resulting in the control of persistent HBV infection in carrier mice.

Despite recent breakthroughs in mRNA technology, DNA vaccines offer unique advantages for therapeutic hepatitis B vaccination. DNA vaccines are thermally more stable and can be delivered as ‘naked’ without the need for complex formulation such as encapsulation into lipid nanoparticles (LNPs). This significantly simplifies their manufacturing process, reduces costs, and facilitates distribution, as DNA vaccines do not require a strict cold chain for transportation and storage as mRNA vaccines do (13). This is particularly important for global administration of the vaccine, also in resource-limited, high-temperature regions where the burden of chronic hepatitis B is highest (1). DNA can be administered repeatedly without the reactogenicity associated with the mRNA platform – chronically infected patients may need several immunizations to break HBV-specific immunotolerance. In addition, a recent report comparing the immunogenicity of COVID-19 vaccines in preclinical animal models found that the mRNA vaccine elicited stronger humoral responses, whereas the DNA vaccine elicited more potent CD8^+^ T cell responses (39).

Inducing potent HBV-specific cellular immune responses is critical for successful therapeutic vaccination, as CD8^+^ T cells are the key effectors in resolving established HBV infection (40, 41). We previously demonstrated that vaccination with recombinant MVA is essential for boosting CD8^+^ T-cell responses and achieving long-term antiviral control of HBV upon priming with adjuvanted recombinant HBsAg and HBcoreAg (7, 12, 27). The antiviral effect of the boost, however, depends strongly on appropriate priming (12). Boosting with the polycistronic MVA-HBVac vector following vaccination with adjuvanted recombinant proteins elicited robust T-cell immunity against S and Core antigens, but only very weak RT(pol)-specific CD8^+^ T cells in naïve C57BL/6J mice. Consistently, the lack of RT(pol) antigen during *TherVacB* priming was insufficient to break RT-specific immune tolerance in HBV-carrier mice persistently replicating HBV of the most clinically relevant genotypes (11). Priming immunization with DNA-HBVac, which expresses the same set of HBV antigens as MVA-HBVac, broadened the vaccine-elicited immunity and allowed the induction of vigorous RT(pol)-specific CD8+ T-cell responses in the spleen and liver. This demonstrated that DNA represents an immunogenic platform that can effectively deliver challenging-to-produce antigens also in the context of therapeutic vaccination.

Although priming with DNA-HBVac induced a potent HBV-specific CD8^+^ T cell response that efficiently suppressed circulating HBeAg levels and reduced the numbers of HBV-positive hepatocytes, in most mice, it failed to achieve sufficient HBsAg-to-anti-HBs seroconversion, which defines a functional HBV cure and constitutes the ultimate goal of effective HBV therapies (42). Only one mouse immunized with the DNA prime-MVA boost regimen, which eliminated persistent HBV infection, was able to clear HBsAg from the serum. Even mice that experienced a reduction of up to 90% in HBV-positive hepatocytes could decrease HBsAg levels by only one log_10_. This strongly supports our previous findings that high levels of anti-HBs induced by *TherVacB* priming with adjuvanted HBsAg are primarily responsible for a decrease in circulating HBsAg (8, 9, 11, 12, 22), and demonstrates that although CD8^+^ T cell-mediated reduction of HBsAg is possible, it is less efficient compared to anti-HBs neutralization. This poses a significant limitation for the development of therapeutic hepatitis B vaccines that rely solely on inducing strong cellular immune responses, particularly since the AAV-HBV mouse model does not support HBV spread (23). On the contrary, *TherVacB*-mediated induction of high anti-HBs titers without efficient CD8^+^ T cell response and elimination of infected hepatocytes could initially reduce circulating HBsAg levels in high-titer HBV-carrier mice. This effect, however, was not sustained, and the rebound in HBsAg was observed shortly thereafter (9). Thus, inducing both humoral and cellular immune responses is needed to achieve immune control over persistent HBV infection.

In contrast to immunization with recombinant proteins, which are potent inducers of humoral immune responses, DNA vaccines express the antigens within the transfected myocytes or antigen-presenting cells (APCs) (43). This results in the presentation of the encoded proteins primarily on MHC-I molecules, leading to vigorous cellular immunity. It has been proposed that antigens released from apoptotic or necrotic cells following DNA vaccination can be captured by professional APCs and presented efficiently on MHC-II molecules, which is essential for antibody development (33, 44). The results of this study, however, demonstrate that only weak anti-HBs and low S-specific IFN-γ^+^ CD4^+^ T cell responses can be primed by a DNA vaccine compared with adjuvanted recombinant proteins. This suggests that the endogenous expression of S protein by DNA and MVA is insufficient to be effectively presented, and consequently, to prime naïve CD4^+^ T cells and B cells. Thus, including secretion motifs (45) that would direct the S protein to the secretory pathway in our DNA-HBvac construct could improve anti-HBs responses. Unexpectedly, both vaccination regimens elicited anti-HBc responses in HBV-naïve and carrier mice, especially when the DNA vaccine was administered at higher doses. HBV Core protein is known to induce anti-HBc in both T-cell-dependent and independent manner (46). Moreover, B-cells function as primary APCs for HBcAg and present the antigen to naïve CD4^+^ T cells more efficiently than dendritic cells and macrophages (47). These unique immunogenic properties of the Core protein may explain why even a small amount of antigen released from DNA-transfected cells *in vivo* can elicit anti-HBc but is insufficient for inducing high titers of anti-HBs. This also supports the notion that providing HBsAg as a recombinant protein alongside the DNA could improve the neutralizing anti-HBs response.

The synergistic effects of simultaneous or sequential DNA and recombinant protein vaccination on improving humoral immunity in mice have been previously reported (48, 49). In our study, sequential regimens, in which DNA and adjuvanted recombinant HBsAg were administered separately over time, proved superior to the simultaneous vaccination. We found that simultaneous priming with DNA and adjuvanted HBsAg indeed induced robust anti-HBs but skewed HBV-specific T-cell responses toward the recombinant protein, inhibiting DNA-mediated immunity in HBV-carrier mice. This implies that mixing DNA with an adjuvant and a protein may directly prevent efficient plasmid delivery to the target cells. Alternatively, a large amount of ready-to-use recombinant proteins can be rapidly processed by APCs via the MHC-II presentation pathway, whereas the DNA vaccine must first express sufficient antigen, predominantly in myocytes and to a lesser extent in APCs, to trigger an immune response (44). By contrast, sequential regimens induced robust humoral and cellular immunity mediated by both vaccine platforms, albeit requiring more immunizations. Sequential immunization initiated with DNA, followed by adjuvanted HBsAg and MVA, elicited a stronger S-specific CD4^+^ T-cell response and highly neutralizing anti-HBs, resulting in superior reductions in HBsAg compared to the group immunized first with adjuvanted HBsAg. Our results indicate that priming a low anti-HBs response with DNA and then enhancing it with adjuvanted HBsAg may result in qualitatively better antibodies with a higher affinity to serum HBsAg than the reverse regimen. Consistently, it has been previously reported that plasmid DNA vaccines are particularly suitable for priming immune responses, as they maintain relatively low-level but stable antigen expression, which may select for immune cells with receptors of higher affinity (44, 50). These S-specific B cells can then be efficiently expanded upon a boost with recombinant protein.

The sequence of immunizations during sequential priming also influenced the specificity of the CD8^+^ T-cell response directed against the S protein. Consistent with previous reports (31, 33), *TherVacB* priming with adjuvanted recombinant proteins elicited S-specific CD8^+^ T cells reactive to the exogenously processed S_208_ epitope, whereas priming with DNA-HBVac elicited endogenously processed S_190_-specific CD8^+^ T cells. A lack of specific CD8^+^ T-cell response directed against the cross-presented S_208_ epitope after DNA priming confirms that HBsAg is insufficiently secreted and cannot effectively enter the MHC-II presentation pathway. However, sequential immunization initiated with recombinant HBsAg resulted in predominant S_208_-specific CD8^+^ T cells, whereas sequential immunization initiated with DNA induced mainly S_190_-specific CD8^+^ T cells, independently of the subsequent immunizations. Since S_190_ is the dominant S-derived peptide presented in AAV-HBV-transduced liver, it is the S_190_-specific CD8^+^ T cells that support the elimination of HBV-positive hepatocytes (51), which may explain the more pronounced antiviral effect observed after immunization with DNA, followed by HBsAg and MVA, compared to the reverse priming regimen. These data demonstrate that the sequence of immunizations, employing diverse antigen carriers in sequential vaccination regimens, can shape the specificity of the desired CD8^+^ T-cell response. Therefore, it may constitute a decisive factor when designing multicomponent heterologous prime-boost strategies.

The AAV-HBV model is characterized by stable, persistent HBV replication and development of strong virus-specific immune tolerance in immunocompetent mice, making it a valuable tool for evaluating immunotherapeutic strategies against chronic hepatitis B. However, HBV cannot directly infect murine hepatocytes and cannot spread within the mouse liver. In contrast to the other mouse HBV models, the formation of covalently closed circular DNA (cccDNA) was observed after AAV-HBV transduction (52). Although in natural infection, cccDNA persists for years in the nuclei of infected hepatocytes and serves as a template for viral transcription, in AAV-HBV mice, the cccDNA pool is lost over time, and its contribution to HBV gene expression is only marginal (53). Thus, the model does not allow for studying the efficacy of vaccination in eliminating cccDNA, which constitutes the ultimate goal of antiviral treatment. In addition, the complete absence of virus-specific immunity in AAV-HBV mice, such as the lack of HBV-specific T-cells or anti-HBcore antibodies, contrasts with that of chronically infected individuals (54).

Taken together, we demonstrate that the synergistic induction of broad and robust virus-specific T-cell responses, along with high titers of functional neutralizing anti-HBs antibodies, is crucial for successful therapeutic vaccination. Although the therapeutic hepatitis B DNA vaccine proved to be safe, easier to produce, and capable of priming a broader T-cell response, it required additional immunizations with adjuvanted protein to elicit the desired anti-HBs response. The order of vaccinations with DNA and recombinant HBsAg during sequential priming determined the neutralization activity of anti-HBs and the specificity of hepatic CD8^+^ T-cell responses, thus significantly influencing the antiviral efficacy of the vaccination. The superior sequential regimen, beginning with DNA followed by adjuvanted HBsAg and MVA, required an increased number of immunizations to match the therapeutic efficacy of ‘classical’ *TherVacB*, which employs only two priming vaccinations with adjuvanted recombinant HBsAg and HBcoreAg, in our preclinical mouse model. Therefore, additional modifications to the DNA backbone and the development of an optimal delivery system are necessary to improve its immunogenicity and enable the successful clinical translation of plasmid DNA vaccines for the treatment of chronic hepatitis B in the future.

## Data Availability

The raw data supporting the conclusions of this article will be made available by the corresponding authors upon reasonable request.
