# Supplementary material for: Therapeutic hepatitis B vaccine employing DNA prime – MVA boost scheme requires additional priming with recombinant HBsAg to elicit an adequate antibody response
Source: Front Immunol. 2026 Mar 12;17:1771887. doi: 10.3389/fimmu.2026.1771887 (PMC13017795; doi:10.3389/fimmu.2026.1771887)
Supplement: Supplementary file 1 [file Table1.docx]

Supplementary Material


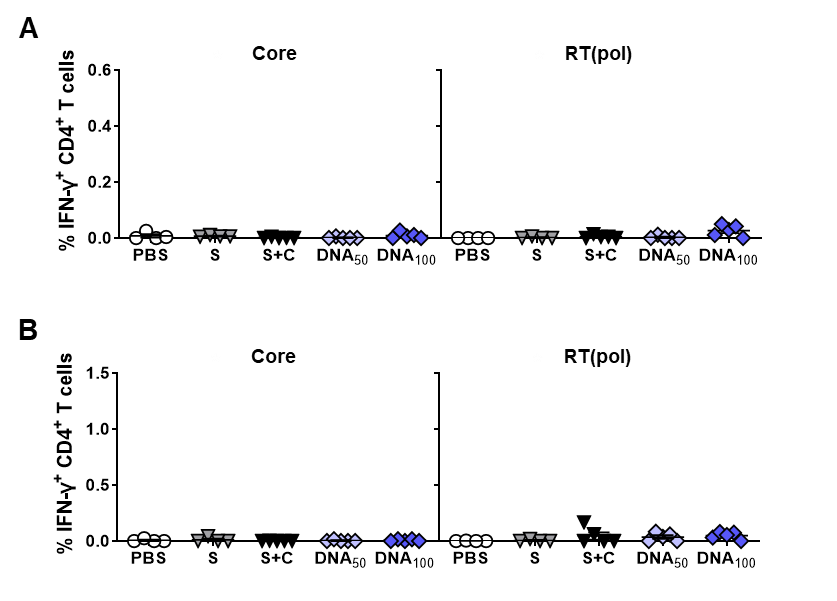


**Supplementary Figure 1. Core- and RT(pol)-specific CD4^+^ T-cell responses induced by DNA prime – MVA boost immunization in HBV carrier mice.** C57BL/6J mice (groups *n*≥ 4) were infected with AAV-HBV and immunized as depicted in Figure 4A. End-point analyses were performed two weeks after MVA boost, at week 6. IFN-γ^+^ CD4^+^ T cells after *ex vivo* stimulation with overlapping HBV core- and RT(pol)-specific peptide pools isolated from **(A)** spleens and **(B)** livers of immunized mice. Mean ± SEM is shown.


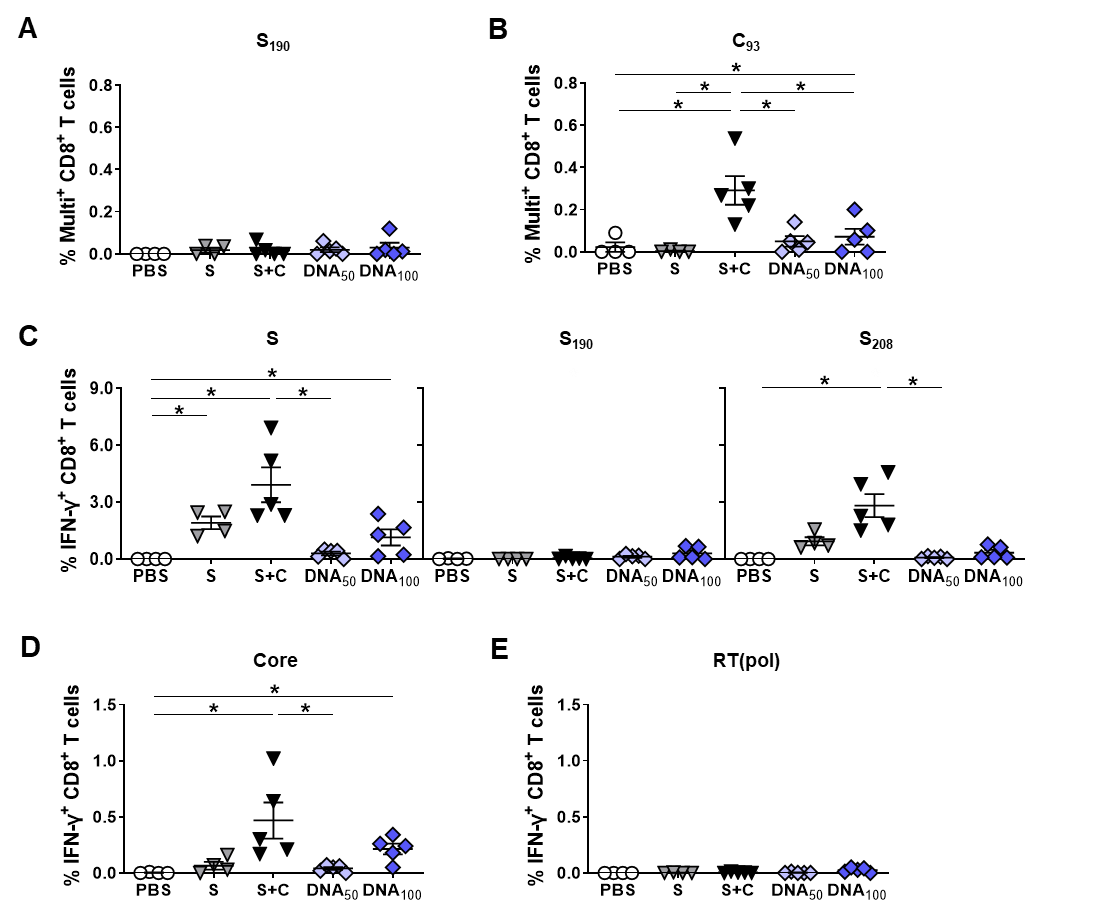


**Supplementary Figure 2. Splenic HBV-specific effector T-cell responses induced by DNA prime – MVA boost immunization in HBV carrier mice.** C57BL/6J mice (groups *n*≥ 4) were infected with AAV-HBV and immunized as depicted in Figure 4A. End-point analyses were performed two weeks after MVA boost, at week 6. Frequencies of **(A)** S- and **(B)** Core-specific CD8^+^ T cells stained positive for S_190_ or C_93_ multimer in the spleen. **(C)** HBV S-specific IFN-γ responses of splenic CD8^+^ T cells determined by ICS after *ex vivo* stimulation with overlapping HBV S-specific peptide pool and single peptides S_190_ and S_208_. HBV **(D)** Core- and **(E)** RT(pol)-specific IFN-γ responses of splenic CD8^+^ T cells determined after *ex vivo* stimulation with overlapping HBV Core- and RT(pol)-specific peptide pools. (B-D) Mean ± SEM is shown. Statistical analysis using nonparametric One-Way ANOVA. *p*-values < 0.05 were considered statistically significant and marked with asterisks (**p*<0.05).


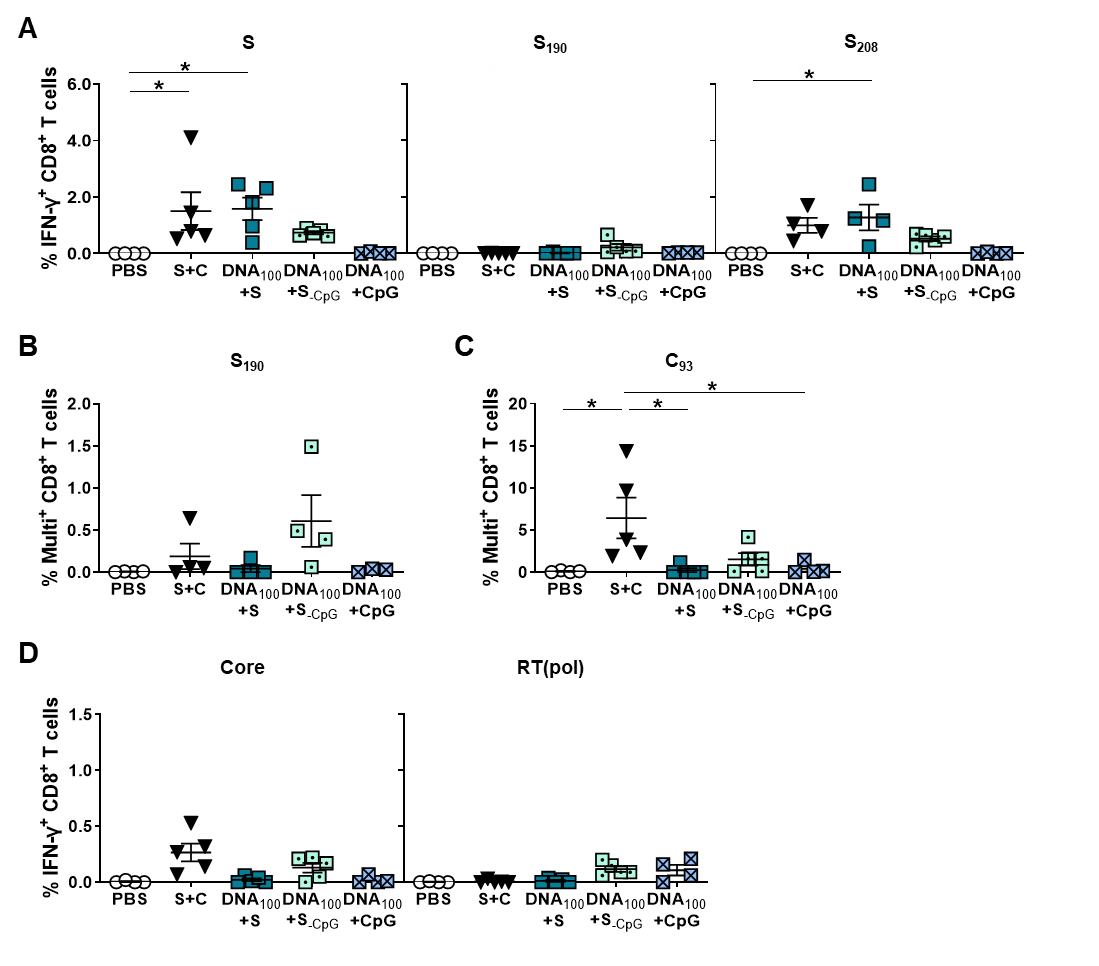


**Supplementary Figure 3. CD8^+^ T-cell responses induced by simultaneous DNA/HBsAg prime – MVA boost immunization in HBV carrier mice.** C57BL/6J mice (groups *n*≥ 4) were infected with AAV-HBV and immunized as depicted in Figure 7A. End-point analyses were performed two weeks after MVA boost, at week 10. **(A)** S-specific IFN-γ responses of splenic CD8^+^ T cells determined by ICS after *ex vivo* stimulation with overlapping HBV S-specific peptide pool and single peptides S_190_ and S_208_. Frequencies of hepatic **(B)** S- and **(C)** Core-specific CD8^+^ T cells detected with S_190_- or C_93_-specific multimers. HBV **(D)** Core- and RT(pol)-specific IFNγ responses of splenic CD8^+^ T cells determined after *ex vivo* stimulation with overlapping Core- and RT(pol)-specific peptide pools. Mean ± SEM is shown. **(A, C)** Statistical analysis using nonparametric One-Way ANOVA. *p*-values < 0.05 were considered statistically significant and marked with asterisks (**p*<0.05).


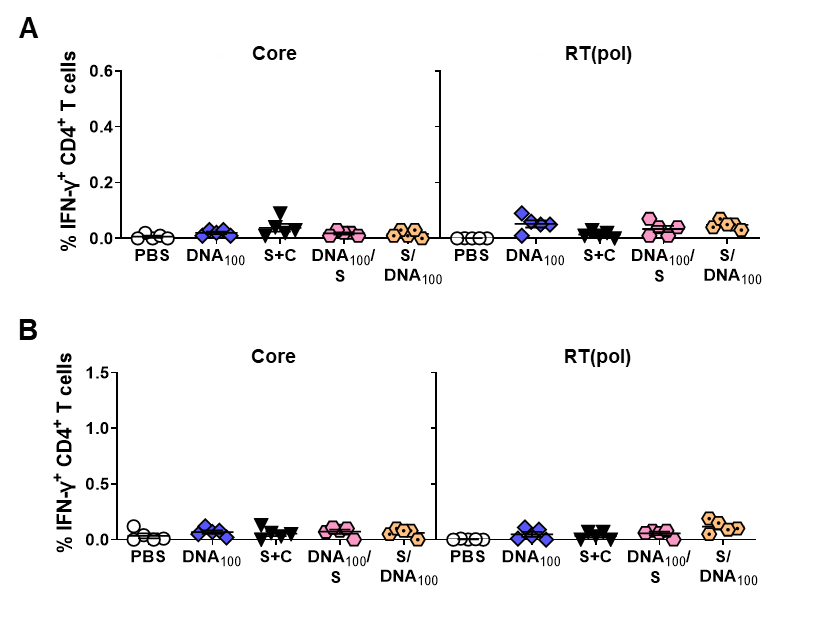


**Supplementary Figure 4. Core- and RT-specific CD4^+^ T-cell responses induced by sequential DNA and HBsAg prime – MVA boost immunization in HBV-carrier mice.** C57BL/6J mice (groups *n*= 5) were infected with AAV-HBV and immunized as depicted in Figure 9A. End-point analyses were performed two weeks after MVA boost, at week 10. **(A-B)** IFN-γ^+^ CD4^+^ T cells after ex vivo stimulation with overlapping HBV core- and RT(pol)-specific peptide pools isolated from **(A)** spleens and **(B)** livers of immunized mice. Mean ± SEM is shown.


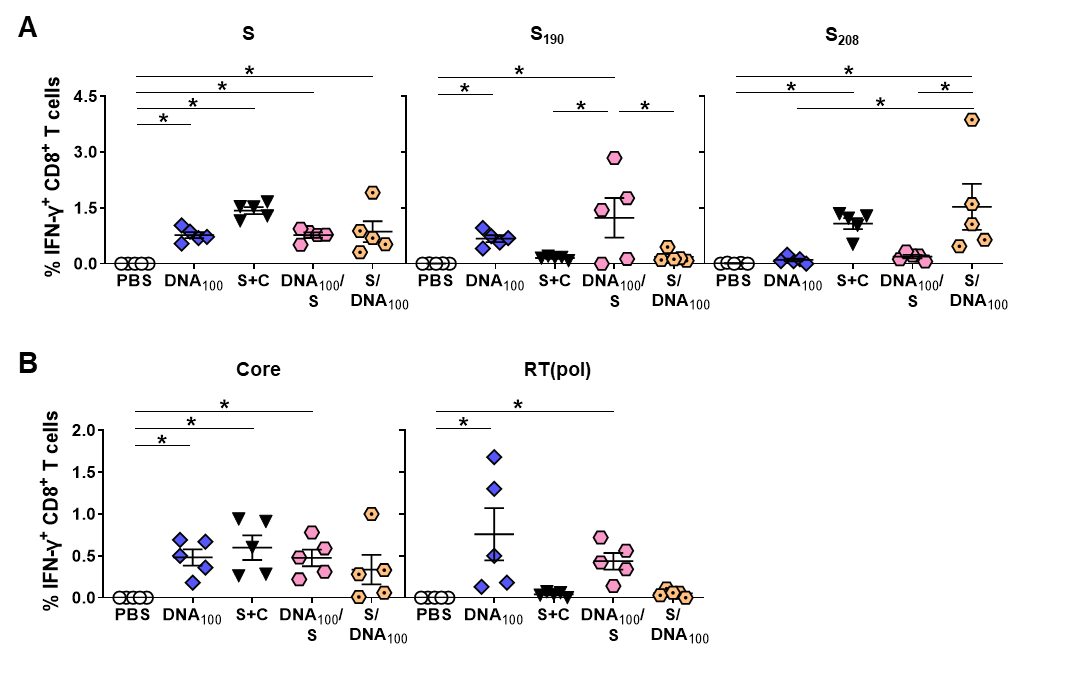


**Supplementary Figure 5. Splenic HBV-specific CD8^+^ T-cell responses induced by sequential DNA and HBsAg prime – MVA boost immunization in AAV-HBV mice.** C57BL/6J mice (groups *n*= 5) were infected with AAV-HBV and immunized as depicted in Figure 9A. End-point analyses were performed two weeks after MVA boost, at week 10. **(A)** S-specific IFN-γ responses of splenic CD8^+^ T cells determined by ICS after *ex vivo* stimulation with overlapping HBV S-specific peptide pool and single peptides S_190_ and S_208_. **(B)** Core- and RT(pol)-specific IFNγ responses of splenic CD8^+^ T cells determined after *ex vivo* stimulation with overlapping Core- and RT(pol)-specific peptide pools. Mean ± SEM is shown. Statistical analysis using nonparametric One-Way ANOVA. *p*-values < 0.05 were considered statistically significant and marked with asterisks (**p*<0.05).

**
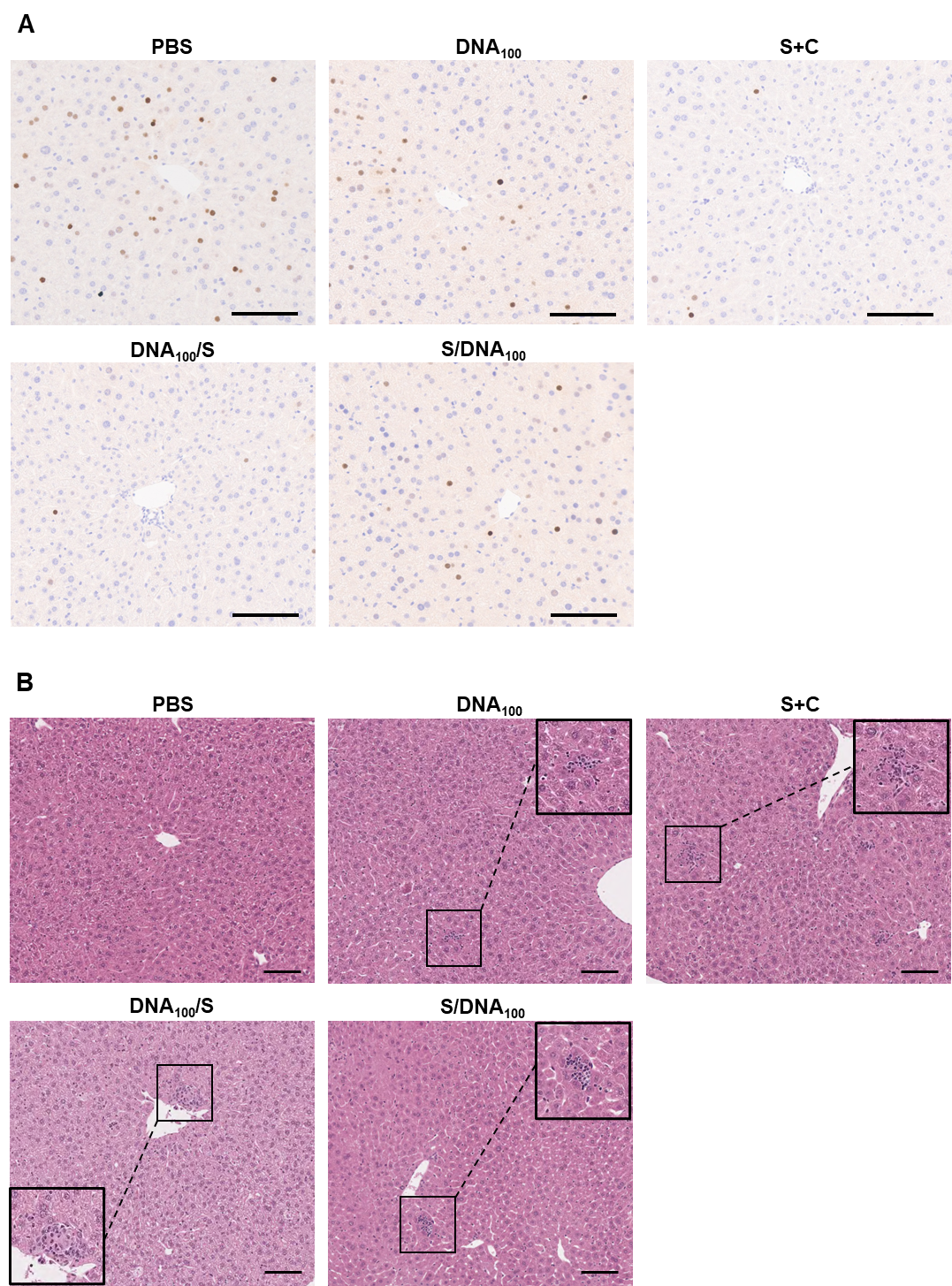
**

**Supplementary Figure 6. Representative histology and immunohistochemistry of HBV carrier mice receiving sequential DNA and HBsAg prime – MVA boost immunization.** C57BL/6J mice (groups *n*= 5) were infected with AAV-HBV and immunized as depicted in Figure 9A. End-point analyses were performed two weeks after MVA boost, at week 10. **(A)** Representative images of liver immunohistochemistry staining for HBcore protein (brown). Scale bars represent 100 μm. **(B)** Representative images of liver sections stained with H&E. Scale bars represent 100 μm, and inlets show magnification of the indicated areas.
